# Firms’ shareholding behavior in green supply chains: Carbon emissions reduction, power structures, and technology spillovers

**DOI:** 10.1016/j.heliyon.2024.e25086

**Published:** 2024-01-22

**Authors:** Wenqiang Li, Juan He, Yangyan Shi

**Affiliations:** a School of Transportation and Logistics, Southwest Jiaotong University, Chengdu, China; b National United Engineering Laboratory of Integrated and Intelligent Transportation, Southwest Jiaotong University, Chengdu, China; c Department of Management, Macquarie Business School, Macquarie University, Sydney, Australia

**Keywords:** Shareholding, Carbon emissions reduction, Equity strategic alliance, Technology spillover, Power structure

## Abstract

Leaders in green supply chains are increasingly focusing on improving strategic synergy with followers through shareholding strategies. By constructing Stackelberg models, we explore the operational mechanisms in two models, manufacturer-led and retailer-led, which have forward and backward shareholding strategies, respectively. Compared with non-shareholding models, we find that the retailer's pricing becomes more sensitive to changes in the market environment after applying shareholding strategies, while the manufacturer's pricing depends on its power status. Interestingly, leaders and entire supply chains prefer shareholding strategies, while followers prefer shareholding strategies in good market environment or in bad market environment with their shares held by leaders below certain thresholds. Moreover, both forward and backward shareholding strategies can effectively promote carbon emissions reduction. Improving manufacturers' technology spillover positively impacts pricing and carbon emissions reduction and profits, and a reasonable shareholding ratio can encourage manufacturers to increase the level of technology spillover. Finally, a two-part tariff contract can effectively coordinate the vertical shareholding supply chain. The results provide decision guidance for managers in applying shareholding strategies to build a strategic alliance to improve firms' economic and environmental performance.

## 1 Introduction

Carbon emissions reduction has become a key metric for assessing the success of sustainable development in a green supply chain and has attracted the attention of international organizations and governments [1,2]. On April 4, 2022, the Intergovernmental Panel on Climate Change released its Sixth Assessment Report, re-emphasizing the importance and urgency of implementing greenhouse gas reduction across all sectors [3].^1^ In China, the government has implemented a series of policies and regulations that provide the external drivers for firms to achieve sustainable development through carbon reductions [4,5].

In addition, consumer preference for green products is an important external driver for firms to achieve sustainable development through carbon reductions. According to a global survey conducted by Accenture, more than 80 % of respondents said they that would consider product greenness when making purchasing decisions.^1^ Carbon Trust's research also shows that over 20 % of consumers are willing to pay a premium for green products.^2^ Driven by the government and consumers, firms are increasingly implementing green development as one of the development strategies to improve their competitiveness. For example, in China, Gree has built a green renewable energy plant in Zhuhai to recycle and reuse resources,^3^ and Haier has built the world's first green recycling connected factory in Qingdao.^4^ Green manufacturing is also being promoted in other industries, including intelligent hardware (Apple), clothing (Converse) and milk (MengNiu) [6]. The existing research's results indicate that designing and producing green products can enhance firms' competitiveness [7].

Based on green manufacturing, firms can further boost their competitiveness by establishing strategic alliances through shareholding, which is a ready-made financial tool that can help them increase cooperation levels. In practice, unlike professional financial investment institutions, core firms in supply chains often invest in upstream or downstream firms for building strategic alliances in order to strengthen control over channels, obtain complementary resources, improve service quality, and enhance supply chain stability. Observing several practical cases, we find that there are the two main forms of equity cooperation, depending on the position of the core firm in green supply chains. In the first, called “forward shareholding,” the upstream manufacturer, who is the leader of a supply chain, holds shares of the downstream retailer. For example, MengNiu, a core company in the dairy supply chain, stabilized its sales channels and improved the efficiency of its fresh milk distribution by holding shares in the downstream retailer Tianxianpei.^5^ The other is known as “backward shareholding.” In this arrangement, the downstream retailer, as the leader of a supply chain, holds shares in the upstream manufacturer. For example, Alibaba, a core firm in the e-commerce supply chain, has chosen to take a 25.25 % stake in an upstream manufacturer, Haier, to stabilize its supply channels and achieve complementary advantages.^6^ Practical cases indicate that the shareholding strategy in green supply chains is frequently utilized to form equity strategic alliances to boost competitiveness, as supported by statistical data showing a yearly increase of almost 25 % in equity strategic alliances since 1987 [8]. However, the failure rate of these alliances is as high as 60%–70 %, the termination rate is almost 50 %, and 30%–70 % of alliances fail to achieve their strategic objectives, causing shareholders to incur economic losses [9,10]. Scholars refer to this phenomenon as an “alliance paradox” [11]. Therefore, the operational mechanism of green shareholding supply chains needs to be explored further in depth.

Green development based on reducing carbon emissions relies on technology innovation. However, industry statistics show that new manufacturing and management technologies are acquired by competitors within 12–15 months due to passive and active technology spillovers from engineers' movements and competitors' “reverse engineering” learning and from the firm's business strategies, respectively [[12], [13], [14]]. Strategic cooperation between firms facilitates the exchange of technology, and the impact of technology exchange within strategic alliances is unknown. Therefore, it is worth exploring what impact manufacturer technology spillovers will have in the green shareholding supply chain.

Based on the above research results and practical cases, we investigate the operational mechanism of a green supply chain in which the core firms apply shareholding strategies to achieve sustainable development while considering the effects of manufacturers' technology spillovers and the supply chain's power structures. Hence, this paper aims to answer the following questions: (1) What are the characteristics of the decision-making behavior of members in green shareholding supply chains? (2) What is the profit distribution mechanism of green shareholding supply chains? What preferences do supply chain members have for shareholding decisions, and does a Pareto region exist to help supply chain members and the entire chain achieve their economic and environmental objectives? (3) How does the power structure affect the decision of the green shareholding supply chain? (4) How does increasing a manufacturer's technology spillover level affect itself and downstream retailers? What shareholding strategies motivate manufacturers to increase their technology spillovers in different power structures? (5) How can members of a strategic alliance further coordinate pecuniary benefits?

Our findings provide decision support for managers of core companies in green supply chains to integrate supply chains through shareholding strategies and provide feasible solutions for governments to realize a low-carbon economy. We find that carbon emissions levels decrease with an increase in the shareholding ratio of the supply chain leaders to the followers. Both the entire chain and the leader can benefit from the shareholding strategy; to do so, the followers need to adjust the proportion of shares held by the leaders according to the specific market environment. Additionally, the manufacturer's technology spillovers positively affect supply chain decisions and profits, and the right equity strategy can lead to higher technology spillovers for manufacturers. Although shareholding strategies can mitigate the double-marginal effect to some extent, they can never eliminate it. The two-part tariff contract can coordinate the members of the green shareholding supply chain.

The contributions of this paper can be summarized as follows. First, we innovatively incorporate both green manufacturing and technology spillovers into a supply chain model with forward and backward shareholding strategies under different power structures. Then we construct a market environment variable and explore ways to contribute to sustainable development in a shareholding supply chain. Relevant research findings complement the green supply chain and equity shareholding research. Second, we explore the impact of manufacturers' technology spillover behavior on decisions and profits while elucidating methods and conditions for increasing manufacturers’ technology spillover rates through shareholding strategies. Third, we present quantitative shareholding methods to benefit all members of green supply chains under different market conditions and further refine the findings of the equity theory in the green supply chain. The research results have guiding significance for firms that intend to improve the efficiency of their supply chain by adopting a vertical shareholding strategy. Our findings can also help the government craft reasonable policies to promote equity cooperation between upstream and downstream enterprises in green supply chains and remove obstacles to the development of a low-carbon economy.

The remainder of the paper is organized as follows. Section 2 reviews the literature on the green supply chain, vertical shareholding, technology spillover, and power structure. Section 3 presents the no-shareholding and shareholding models and the equilibrium analysis. Section 4 analyzes the impact of vertical shareholding. Section 5 investigates the influence of technology spillovers. In Section 6, we adopt a two-part tariff contract to coordinate further the vertical shareholding supply chain. Finally, Section 7 presents the paper's conclusions and offers suggestions for future research directions.

## 2 Literature review

Summarizing the existing research literature, this paper is relevant to the following four research areas: (1) green supply chain, (2) vertical shareholding, (3) technology spillover, and (4) power structure.

### 2.1 Green supply chain

Environmental change poses a vast threat to humanity. Therefore, how to achieve sustainable development has always been a hot topic in the research area [15]. The research on green supply chains has also achieved fruitful results. One of the important research directions is to focus on decision making to maximize the economic and environmental benefits of green supply chains. For example, Shahzad et al. (2020) examined how organizational compatibilities affect green supply chain management efforts (GSCM) and their impact on organizational performance, showing that organizational compatibilities have a positive effect on promoting GSCM efforts [16]. Jin et al. (2021) found that managers' optimistic bias will reduce enterprises' R&D investment in green products, but their optimism is beneficial to all stakeholders only when certain conditions are met [17]. Small and medium-sized enterprises face huge cost pressure relating to carbon emissions reduction, Wang et al. (2021) studied the impact of supply chain leaders' altruistic preferences on carbon emissions reduction [18]. Daryanto et al. (2019) established a triple supply chain considering carbon emissions reduction and item deterioration, and the results show that centralized decision making in the supply chain is beneficial in reducing supply chain costs and carbon emissions [19]. To verify the impact of the carbon tax policy on supply chain decision making, Luo et al. (2022) developed four game models and found that the carbon tax policy can effectively improve the carbon emissions reduction level of supply chain [20]. Abbas et al. (2023) examined the intricate connection between information technology capabilities, green supply chain integration, and green innovation on firms’ performance. They discovered that the interrelation between different aspects of green supply chain integration and performance is mediated by green process innovation and green product innovation [21]. Other studies on this issue also consider institutional pressure [22], financing strategy [23], traceability [24], and big data analytical capabilities [25].

The other important research direction is cooperation and coordination between upstream and downstream firms in green supply chains [26]. For example, Klassen et al. (2003) found that strengthening the cooperation between enterprises in green supply chains can effectively promote manufacturers to increase their R&D investment in environmental protection [27]. Jira and Toffel (2013) used public data to analyze the factors that affect the cooperation between upstream and downstream enterprises in green supply chains. The results show that upstream and downstream companies are more inclined to cooperate when the industry in which the supply chain is located is more profitable and has environmental protection policy supervision [28]. Dai et al. (2017) discussed the impact of cartelization and cost-sharing contracts on green supply chain decision making. They claimed that cartelization is beneficial to upstream companies, while cost-sharing contracts are beneficial to both upstream and downstream companies [29]. Hafezalkotob (2017) considered the influence of government financial intervention on green supply chains. He discovered that strengthening cooperation between upstream and downstream enterprises in supply chains can enable them to meet policy requirements better [30]. Under the cap-and-trade policy, Kuiti et al. (2022) sequentially analysed the impact of the two-part tariff contract, the corporate social responsibility effort sharing contract and the cost-sharing contract on supply chain decision making in the two cases of a centralized model and a decentralized model [31]. Peng et al. (2023) investigated a trade-off problem between self-produced and purchased hydrogen quantities to hydrogen refuelling stations, showing that hydrogen prices, carbon trading prices, and carbon emissions reduction efficiency all influence the optimal decisions of production plans and carbon emissions reduction. The results show that the cap-and-trade policy prompts companies to increase their R&D investment in carbon emissions reduction, and consumers face higher prices when the supply chain adopts the cost-sharing contract [3].

Different from prior research, we introduce shareholding strategies in green supply chains. This novel cooperation method investigates the impact of shareholding on pricing and profits, leading to compelling new findings.

### 2.2 Vertical shareholding

Academics and business people have always valued the shareholding strategy, and practice has proved that it can effectively improve supply chain efficiency [32]. Some scholars have explored how shareholding affects output and demand in supply chains. For example, Flath (1989) found that vertical shareholding can increase the output of supply chains [33]. Greenlee et al. (2006) discovered that demand increases when downstream firms hold shares in upstream firms [34].

Some scholars specifically explored the influence of shareholding on supply chain members’ decisions. For example, Qi et al. (2016) explored the decision-making problem of a supply chain, specifically in the case of multiple downstream enterprises investing in a shared supplier. They found that invested enterprises tend to discourage competitors from also investing in the same supplier [35]. Hunold and Stahl (2016) studied the backward shareholding model and found that the decision-making of upstream enterprises can be influenced by the shareholding strategy [36]. Gaigné et al. (2018) revealed the regular pattern that the more productive manufacturers are, the more they tend to take shares in downstream companies [37]. Fu et al. (2018) investigated the supply chain model with vertical shareholding and found that strengthening the shareholding of downstream firms in upstream firms is conducive to improving product quality and reducing the product price [38]. Additionally, Zhang et al. (2021) conducted research on the equity financing mechanism within a supply chain and found that the equity dividend ratio has an impact on the profit of the retailer [39].

Other scholars focused on the role of cross-shareholding in supply chains. For example, Chen et al. (2017) examined the role of cross-shareholding in push-and-pull supply chain systems and found that the power structures of supply chains determine whether the pull or the push supply chain performs better [40]. Additionally, Ren et al. (2021) concentrated on the selection of shareholding strategies and found that upstream and downstream enterprises in supply chains can achieve a Pareto improvement with the cross-shareholding strategy [41]. Fu and Ma (2019) investigated the role of cross-shareholding in supply chains. They discovered that to achieve a win-win situation for all members of the supply chains, one member should first operate at a loss and then make up for the loss through the profit earned from the shareholding [42]. Furthermore, Xia et al. (2021) analysed the impact of cross-shareholding on green supply chain, showing that the effect of shareholding on carbon emissions reduction is related to power structures [43].

Different from the previous study, we aim to investigate how core firms within green supply chains integrate with their partners. Additionally, we will examine the impact of manufacturers’ green responsibility and technology spillovers, which provide some significant insights. Our research will amend certain existing conclusions on equity cooperation as discussed in the previous study.

### 2.3 Technology spillovers

Technology spillover effects are common in supply chains, and some companies are very vigilant about this phenomenon and actively set up obstacles to maintain their relative technological advantages [44]. However, some companies are optimistic about technology spillovers and take the initiative to help relative companies in the supply chain to improve their technical level and production efficiency. For example, Toyota uses its own technological advantages to help upstream and downstream enterprises improve their technological level [13]. Against the background of economic globalization, multinational enterprises are developing rapidly. Wang and Wu (2016) found that the technology spillovers brought by foreign investment are very beneficial to domestic enterprises and promote their innovation [45]. The above papers explore the effects of technology spillovers from an empirical perspective, whereas some scholars explore the influence of technology spillovers from a theoretical perspective. For example, Ge et al. (2014) found that the impact of technology spillovers on enterprises is related to whether there is cooperation between enterprises [46]. Byun et al. (2021) investigated the impact of technology spillovers on the form of firm innovation, and the results showed that technology spillovers are positively correlated with the number of outcomes of firms’ overall innovation but make firms focus on incremental innovation rather than breakthrough innovation [47]. Chen et al. (2019) took the two-echelon green supply chain as their research object to study the impact of corporate R&D investment on decision making and profits. They found that the efficiency of enterprise R&D investment and technology spillovers jointly affect the profit of an enterprise [48]. Due to the high risk of R&D investment, Niu and Shen (2022) investigated whether manufacturers are willing to increase their R&D investment in the face of the technology spillover effect with different learning abilities. They found that technology spillovers have an important impact on corporate R&D decisions when the probability of R&D success is high [49].

However, previous research has overlooked the interplay between technology spillovers and stockholding strategies. In contrast, this study investigates the effect of technology spillovers on decision-making for green supply chains and the impact of shareholding decisions on technology spillovers.

### 2.4 Power structure

Research regarding power structures has been valued by theoretical researchers and practitioners. Some scholars focus on the effects of power structure on the operational mechanism of supply chains. For example, Yao and Liu (2005) explored the influence of the power structure on the pricing decisions of different channels [50]. Agi and Yan (2020) analysed the influence of the power structure on the operation of green supply chains, and the results show that manufacturer-led supply chains have more advantages in reducing fixed costs than retailer-led supply chains [51]. Others focus on investigating the effects of power structure on strategic choice of supply chain members. For example, Pan et al. (2010) investigated the influence of the supply chain power structure on contract selection, showing that it is more beneficial for the manufacturer to choose the revenue-sharing contract when the manufacturer dominates the supply chain [52]. In the context of the carbon tax policy's implementation, Meng et al. (2018) explored the impact of different power structures on enterprises' choice of low-carbon products. In the Nash game, two competing firms will make the same product selection decision irrespective of the carbon tax [53]. In the Stackelberg game, the firms' product choices are related to the carbon tax. Additionally, other scholars considered the influence of the supply chain power structure and channel selection on decision making [54,55]. However, the effects of power structure on shareholding and technology spillovers in green supply chains have not been investigated in their research.

In practice, vertical shareholding, technology spillovers, and power structures have important influences on supply chains’ decisions, but the previous studies on green supply chains do not simultaneously consider the roles of these three factors nor adequately explore what effects these three factors have on each other. To fill the existing research gap, this paper explores, under the two power structures, the specific ways in which core supply chain firms apply shareholding strategies to integrate resources to achieve sustainable development and thoroughly investigates the interaction between shareholding strategies and technology spillovers. Table 1 shows the differences between this paper and other studies, and list our major contributions.

**Table 1:** Summary of the literature review.

Articles	Green supply chain	Vertical shareholding	Technology spillover	Power structure
Luo et al. (2022)	**✓**			
Hafezalkotob (2017)	**✓**			
Chen et al. (2017)	**✓**			**✓**
Kuiti et al. (2022)	**✓**			
Ren et al. (2021)	**✓**	**✓**		
Xia et al. (2021)	**✓**			**✓**
Chen et al. (2019)	**✓**		**✓**	**✓**
Niu et al. (2022)			**✓**	
This paper	**✓**	**✓**	**✓**	**✓**

## 3 Models and equilibrium analysis

We construct two green supply chain models, each consisting of a manufacturer and a retailer. When the manufacturer (he) is the supply chain leader, the model is called the MD model, which includes the manufacturer-dominated non-shareholding model and the shareholding model, and when the retailer (she) is the supply chain leader, the model is called the RD model, which includes the retailer-dominated non-shareholding model and the shareholding model. We assume that both the manufacturer and the retailer are perfectly rational and aim to maximize their own profits. Driven by regulations and consumer pressures, many companies are investing in reducing carbon emissions. We use e to represent the manufacturers’ carbon emissions reduction level, and the carbon emissions reduction cost is $\frac{1}{2}ze^{2}$ [18,43], which has a quadratic form and implies a convex increasing cost in terms of the carbon emissions reduction level. In addition, z represents the unit carbon emissions reduction cost, and a smaller z indicates that the manufacturer has greater emissions reduction efficiency [31,48].

In green supply chains, the manufacturer can adjust his technology spillover rate based on corporate strategies, which can improve the carbon emissions reduction efficiency. We use θ to denote the technology spillover rate of the manufacturer, and θe is the increment in the carbon emissions reduction level of the entire supply chain caused by the technology spillover of the manufacturer. R&D requires significant investment, and the opportunity cost loss to the manufacturer from technology spillovers is denoted by $\frac{1}{2}\theta ze^{2}$ [56,57]. Furthermore, we assume that consumers have the same preference for eco-friendly products and are price sensitive. Therefore, the market demand faced by the retailer is q=D−εp+λ(1+θ)e, as frequently cited in previous literature [31,41,42,58]. Without carbon emissions reduction investment, there is q=D−εp>0. Table 2 shows the specific explanations of relevant decision variables and parameters.

**Table 2:** Parameters and variables.

Decision variables	Descriptions
p	The selling price (p>w)
w	The wholesale price (w>c)
e	The carbon emission reduction level (e>0)
**Parameters**	
c	The unit production cost (c>0)
D	The maximum market demand (D>0)
ε	The price sensitivity of the consumer (ε>0)
λ	The carbon emission reduction sensitivity of the consumer (λ>0)
z	The unit carbon emission reduction cost (z>0)
θ	The technology spillover rate (0≤θ≤1)
$\varphi_{m}$	The proportion of the retailer's shares held by the manufacturer $\left(0<\varphi_{m}<50\%\right)$
$\varphi_{r}$	The proportion of the manufacturer's shares held by the retailer $\left(0<\varphi_{r}<50\%\right)$
$\Pi_{m}$	The manufacturer's profit
$\Pi_{r}$	The retailer's profit

### 3.1 Decentralized supply chain model without shareholding

We describe the decision-making sequences of the non-shareholding model under different power structures.

**Fig. 1:**
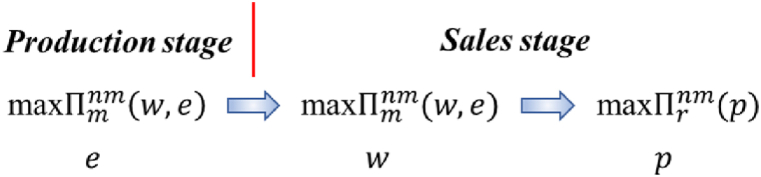
The decision sequence of the nm model.

**Fig. 2:**
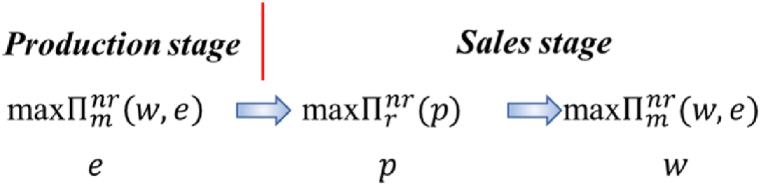
The decision sequence of the nr model.

**The manufacturer-dominated non-shareholding supply chain**: In the production stage, the manufacturer decides the carbon emissions reduction level e according to the market demand and his own technical level. In the sales stage, the manufacturer first decides the wholesale price w, and then the retailer decides the selling price p. Therefore, the decision sequence for the nm model is as follows (nm stands for the manufacturer-dominated non-shareholding model, and see Fig. 1):

**The retailer-dominated non-shareholding supply chain**: In the production stage, the manufacturer decides the carbon emissions reduction level e. In the sales stage, the retailer first decides the selling price p, and then the manufacturer decides the wholesale price w. Therefore, the decision sequence for the nr model is as follows (nr stands for the retailer-dominated non-shareholding model, and see Fig. 2):

In non-shareholding models, the profit functions for manufacturers and retailers are:

(1) $$\Pi_{m}^{ni}\left(w,e\right)=\left(w-c\right)\left[D-\varepsilon p+\lambda \left(1+\theta \right)e\right]-\frac{1}{2}z\left(1+\theta \right)e^{2}$$

(2) $$\Pi_{r}^{ni}\left(p\right)=\left(p-w\right)\left[D-\varepsilon p+\lambda \left(1+\theta \right)e\right]$$

where i=m,r. Table 3 shows the equilibrium analysis results of the manufacturer-led and the retailer-led non-shareholding models based on Equations (1), (2). Appendix A provides the detailed solution process.

**Table 3:** Equilibrium analysis.

Models		MD Model(i = m)	RD Model(i = r)
Non-shareholding	$w^{ni}$	$c+\frac{2z\left(D-\varepsilon c\right)}{4\varepsilon z-\lambda^{2}\left(1+\theta \right)}$	$c+\frac{2z\left(D-\varepsilon c\right)}{8\varepsilon z-\lambda^{2}\left(1+\theta \right)}$
	$e^{ni}$	$\frac{\left(D-\varepsilon c\right)\lambda }{4\varepsilon z-\lambda^{2}\left(1+\theta \right)}$	$\frac{\left(D-\varepsilon c\right)\lambda }{8\varepsilon z-\lambda^{2}\left(1+\theta \right)}$
	$p^{ni}$	$c+\frac{3z\left(D-\varepsilon c\right)}{4\varepsilon z-\lambda^{2}\left(1+\theta \right)}$	$c+\frac{6z\left(D-\varepsilon c\right)}{8\varepsilon z-\lambda^{2}\left(1+\theta \right)}$
	$\Pi_{m}^{ni}$	$\frac{z{\left(D-\varepsilon c\right)}^{2}}{2\left[4\varepsilon z-\lambda^{2}\left(1+\theta \right)\right]}$	$\frac{z{\left(D-\varepsilon c\right)}^{2}}{2\left[8\varepsilon z-\lambda^{2}\left(1+\theta \right)\right]}$
	$\Pi_{r}^{ni}$	$\frac{\varepsilon z^{2}{\left(D-\varepsilon c\right)}^{2}}{{\left[4\varepsilon z-\lambda^{2}\left(1+\theta \right)\right]}^{2}}$	$\frac{8\varepsilon z^{2}{\left(D-\varepsilon c\right)}^{2}}{{\left[8\varepsilon z-\lambda^{2}\left(1+\theta \right)\right]}^{2}}$
Shareholding	$w^{si}$	$c+\frac{2z\left(1-\varphi_{m})(D-\varepsilon c\right)}{2\varepsilon z\left(2-\varphi_{m}\right)-\lambda^{2}\left(1+\theta \right)}$	$c+\frac{\left(2-\varphi_{r})z(D-\varepsilon c\right)}{2\varepsilon z{\left(2-\varphi_{r}\right)}^{2}-\lambda^{2}\left(1+\theta \right)}$
	$e^{si}$	$\frac{\left(D-\varepsilon c\right)\lambda }{2\varepsilon z\left(2-\varphi_{m}\right)-\lambda^{2}\left(1+\theta \right)}$	$\frac{\left(D-\varepsilon c\right)\lambda }{2\varepsilon z{\left(2-\varphi_{r}\right)}^{2}-\lambda^{2}\left(1+\theta \right)}$
	$p^{si}$	$c+\frac{z\left(3-{2\varphi }_{m})(D-\varepsilon c\right)}{2\varepsilon z\left(2-\varphi_{m}\right)-\lambda^{2}\left(1+\theta \right)}$	$c+\frac{\left(3-2\varphi_{r}\right)\left(2-\varphi_{r}\right)z\left(D-\varepsilon c\right)}{2\varepsilon z{\left(2-\varphi_{r}\right)}^{2}-\lambda^{2}\left(1+\theta \right)}$
	$\Pi_{m}^{si}$	$\frac{z{\left(D-\varepsilon c\right)}^{2}}{2\left[2\varepsilon z\left(2-\varphi_{m}\right)-\lambda^{2}\left(1+\theta \right)\right]}$	$\frac{\left(1-\varphi_{r}\right)z{\left(D-\varepsilon c\right)}^{2}}{2\left[2\varepsilon z{\left(2-\varphi_{r}\right)}^{2}-\lambda^{2}\left(1+\theta \right)\right]}$
	$\Pi_{r}^{si}$	$\frac{\left(1-\varphi_{m}\right)\varepsilon z^{2}{\left(D-\varepsilon c\right)}^{2}}{{\left[2\varepsilon z\left(2-\varphi_{m}\right)-\lambda^{2}\left(1+\theta \right)\right]}^{2}}$	$\frac{z{\left(D-\varepsilon c\right)}^{2}\left[2\varepsilon z{\left(2-\varphi_{r}\right)}^{3}-\varphi_{r}\lambda^{2}\left(1+\theta \right)\right]}{{2\left[2\varepsilon z{\left(2-\varphi_{r}\right)}^{2}-\lambda^{2}\left(1+\theta \right)\right]}^{2}}$

The profits of the decentralized supply chains in the non-shareholding models are:

$${\Pi_{t}^{ni}\left(w,e,p\right)=\Pi }_{m}^{ni}\left(w,e\right)+\Pi_{r}^{ni}\left(p\right),\text{where}\;i=m,r$$

### 3.2 Decentralized supply chain model with shareholding

To ensure that the enterprise has independent decision-making power, this paper considers the situation of the same shares and the same rights, and the shareholding ratio does not exceed 50 %. This subsection investigates shareholding supply chains and describes the decision-making sequence of the shareholding models under different power structures.

**The manufacturer-dominated shareholding supply chain**: The manufacturer, as the leader, holds $\varphi_{m}$ shares in the retailer and forms an equity strategic alliance in the green supply chain, and $\varphi_{m}$ of the retailer's profit will be shared by the manufacturer. The decision sequence of the sm model is the same as that of the nm model (sm stands for the manufacturer-dominated shareholding model), and the profit functions of the manufacturer and the retailer are:

(3) $$\Pi_{m}^{sm}\left(w,e\right)=\left(w-c\right)\left[D-\varepsilon p+\lambda \left(1+\theta \right)e\right]-\frac{1}{2}z\left(1+\theta \right)e^{2}+\varphi_{m}\left(p-w\right)\left[D-\varepsilon p+\lambda \left(1+\theta \right)e\right]$$

(4) $$\Pi_{r}^{sm}\left(p\right)=\left(1-\varphi_{m}\right)\left(p-w\right)\left[D-\varepsilon p+\lambda \left(1+\theta \right)e\right]$$

**The retailer-dominated shareholding supply chain**: The retailer, as the leader, holds $\varphi_{r}$ shares in the manufacturer and forms an equity strategic alliance in the supply chain, and $\varphi_{r}$ of the manufacturer's profit and carbon emissions reduction cost will be shared by the retailer. The decision sequence of the sr model is the same as that of the nr model (sr stands for the retailer-dominated shareholding model), and the profit functions of the manufacturer and the retailer are:

(5) $$\Pi_{m}^{sr}\left(w,e\right)=\left(1-\varphi_{r}\right)\left(w-c\right)\left[D-\varepsilon p+\lambda \left(1+\theta \right)e\right]-\frac{1}{2}\left(1-\varphi_{r}\right)z\left(1+\theta \right)e^{2}$$

(6) $$\Pi_{r}^{sr}\left(p\right)=\left(p-w\right)\left[D-\varepsilon p+\lambda \left(1+\theta \right)e\right]+\varphi_{r}\left(w-c\right)\left[D-\varepsilon p+\lambda \left(1+\theta \right)e\right]-\frac{1}{2}\varphi_{r}z\left(1+\theta \right)e^{2}$$

Based on Equations (3), (4), the optimal solution of the manufacturer-dominated shareholding model is determined. Using Equations (5), (6), the retailer-dominated shareholding model is solved. Table 3 presents the equilibrium analysis results, and the detailed solution process is shown in Appendix A**.**

In the subsequent analysis, we have $u_{e}=\frac{\lambda^{2}\left(1+\theta \right)}{2\varepsilon z}$. Obviously, $u_{e}$ increases with the increase in consumers' sensitivity to carbon emissions reduction λ and the manufacturer's technological spillover rate θ but decreases with the increase in the price sensitivity of the customer ε and the unit carbon emissions reduction cost z. From the point of view of manufacturers and retailers, the larger the $u_{e}$, the better. Therefore, we define $u_{e}$ as the market environment faced by manufacturers and retailers, as commonly presumed in the abundant green supply chain papers [46,48]. In contrast to existing analytical methods, we examine the operating mechanism of equity strategic alliances in green supply chains in different market environments with the help of the market environment variable. Under the following assumptions, the range of values of the market environment variable indicates the extent to which various models have adapted to the market [46,48].

To guarantee the existence of all decisions in MD models, we have the following hypothesis:

$${0<u}_{e}<\frac{3}{2}$$

Similarly, we have the following hypothesis in RD models:

$${0<u}_{e}<\frac{9}{4}$$

In the subsequent analysis, we illustrate the conclusions of certain propositions through graphic representations in a subsequent analysis. The data in the numerical simulation only need to be consistent with the above assumptions. Actual case data can also serve as a substitute, and these cases easily satisfy the assumptions of this paper and do not affect the validity of the conclusions. For the purpose of simplification, detailed data used in this paper have not been listed due to the large amount utilized. Additionally, the profits of the decentralized supply chains in the shareholding models are:

$${\Pi_{t}^{si}\left(w,e,p\right)=\Pi }_{m}^{si}\left(w,e\right)+\Pi_{r}^{si}\left(p\right),\text{where}\;i=m,r$$

## 4 Effects of shareholding

Based on the equilibrium analysis results, we analyze the impact of shareholding on supply chain members’ decisions and profits. Appendix B provides the proofs of this section.

### 4.1 The effects of shareholding on decisions

Proposition 1 (1) In the MD model, (i) $e^{\text{sm}}>e^{\text{nm}}$ and (ii) $q^{\text{sm}}>q^{\text{nm}}$.

(2) In the RD model, (i) $e^{\text{sr}}>e^{\text{nr}}$ and (ii) $q^{\text{sr}}>q^{\text{nr}}$.

In both the MD and RD models, the carbon emissions reduction levels and order quantities in shareholding models are always greater than those in the non-shareholding models. Proposition 1 indicates that it is beneficial for leaders to strengthen the integration of the supply chain to reduce more carbon emissions, and leaders can effectively improve the cooperation level with their partners by holding followers’ shares under different power structures.

Proposition 2 (1) In the MD model, (a) when $0<u_{e}<1$, then $w^{\text{sm}}<w^{\text{nm}}$; (b) when $u_{e}=1$, then $w^{\text{sm}}=w^{\text{nm}}$; and (c) when $1<u_{e}<\frac{3}{2}$, then $w^{\text{sm}}>w^{\text{nm}}$.

(2) In the RD model, $w^{\text{sr}}>w^{\text{nr}}$.

In the MD model, when the market environment is relatively bad ($0<u_{e}<1$), $w^{\text{sm}}$ is always smaller than $w^{\text{nm}}$ and vice versa. Interestingly, $w^{\text{sm}}$ is independent of $\varphi_{m}$ when $u_{e}=1$. In the RD model, the manufacturer’s wholesale price in the shareholding model is always greater than that in the non-shareholding model. If the retailer’s shareholding ratio $\varphi_{r}$ to the manufacturer increases, the manufacturer will increase the wholesale price $w^{\text{sr}}$. This is because $\varphi_{r}$ of the profit of the manufacturer is divided by the retailer, and the manufacturer needs to increase the unit price of the product to ensure its own profit. To sum up, the pricing strategies of manufacturers in the shareholding supply chains are influenced by the power structures.

Proposition 3 (1) In the MD model, (a) when $0<u_{e}<\frac{1}{2}$, then $p^{\text{sm}}<p^{\text{nm}}$; (b) when $u_{e}=\frac{1}{2}$, then $p^{\text{sm}}=p^{\text{nm}}$; and (c) when $\frac{1}{2}<u_{e}<\frac{3}{2}$, then $p^{\text{sm}}>p^{\text{nm}}$.

(2) In the RD model, (a) when $0<u_{e}\leq \frac{1}{2}$, then $p^{\text{sr}}<p^{\text{nr}}$; (b) when $\frac{1}{2}<u_{e}<\frac{4}{7}$, if $0<\varphi_{r}<\varphi_{r1}$, then $p^{\text{sr}}<p^{\text{nr}}$; if $\varphi_{r1}\leq \varphi_{r}<50\%$, then $p^{\text{sr}}\geq p^{\text{nr}}$; and (c) when $\frac{4}{7}\leq u_{e}<\frac{9}{4}$, then $p^{\text{sr}}>p^{\text{nr}}$, where $\varphi_{r1}=\frac{4-7u_{e}}{2\left(1-u_{e}\right)}$.

Proposition 3 shows that the selling price in the shareholding supply chain is affected by the market environment $u_{e}$. In the MD model, when $u_{e}$ is small (large), $p^{\text{sm}}$ is smaller (larger) than $p^{\text{nm}}$. Interestingly, $p^{\text{sm}}$ is independent of $\varphi_{m}$ when $u_{e}=\frac{1}{2}$, which makes $p^{\text{sm}}$ is equal to $p^{\text{nm}}$. In the RD model, as the leader of the supply chain, the retailer’s pricing is relatively complex. Compared with the pricing decision in the non-shareholding model, the retailer in the shareholding supply chain has lower (higher) prices when the market environment is relatively bad (good). What we find interesting is that when $u_{e}$ is between $\frac{1}{2}$ and $\frac{4}{7}$, there is a critical value of $\varphi_{r1}$ for the retailer’s shareholding rate $\varphi_{r}$, such that when $\varphi_{r}$ is less than (greater than or equal to) $\varphi_{r1}$, the selling price of the shareholding model is less than (greater than or equal to) that of the non-shareholding model.

To sum up, when the market environment changes, retailers in the shareholding supply chains are more proactive in their pricing strategies. The results show that when supply chain leaders apply a shareholding strategy to followers, retailers make more active pricing decisions to address the impact of market environment changes on their profits.

### 4.2 The effects of shareholding on profits

Proposition 4 (1) In the MD model, $\Pi_{t}^{\text{sm}}\left(w,e,p\right)>\Pi_{t}^{\text{nm}}\left(w,e,p\right)$ and $\frac{\partial \Pi_{t}^{\text{sm}}\left(w,e,p\right)}{\partial \varphi_{m}}>0$.

(2) In the RD model, $\Pi_{t}^{\text{sr}}\left(w,e,p\right)>\Pi_{t}^{\text{nr}}\left(w,e,p\right)$ and $\frac{\partial \Pi_{t}^{\text{sr}}\left(w,e,p\right)}{\partial \varphi_{r}}>0$.

Proposition 4 shows that, whether in a manufacturer-led or in a retailer-led supply chain, when the leader adopts the shareholding strategy for the follower, the overall profit of the chain will be higher than that of the chain without the shareholding strategy. Moreover, the advantage of a shareholding supply chain is more obvious with the increase in the shareholding proportion. From the perspective of the entire supply chain, it is beneficial for the leader to apply a shareholding strategy to the follower. The shareholding strategy helps to strengthen the linkage between upstream and downstream firms and assists firms in making decisions that are beneficial to the aggregate pecuniary benefit.

Proposition 5 (1) In the MD model, (i) $\Pi_{m}^{\text{sm}}\left(w,e\right)>\Pi_{m}^{\text{nm}}\left(w,e\right)$; and (ii) (a) when $0<u_{e}<\frac{2-\sqrt{2}}{2}$, if $0<\varphi_{m}<\varphi_{m1}$, then $\Pi_{r}^{\text{sm}}\left(p\right)>\Pi_{r}^{\text{nm}}\left(p\right)$; if $\varphi_{m1}\leq \varphi_{m}<50\%$, then $\Pi_{r}^{\text{sm}}\left(p\right)\leq \Pi_{r}^{\text{nm}}\left(p\right)$; (b) when $\frac{2-\sqrt{2}}{2}\leq u_{e}<\frac{3}{2}$, then $\Pi_{r}^{\text{sm}}\left(p\right)>\Pi_{r}^{\text{nm}}\left(p\right)$, where $\varphi_{m1}=1-{\left(1-u_{e}\right)}^{2}$.

(2) In the RD model, (i) (a) when $0<u_{e}<\frac{1}{2}$, if $0<\varphi_{r}<\varphi_{r2}$, then $\Pi_{m}^{\text{sr}}\left(w,e\right)>\Pi_{m}^{\text{nr}}\left(w,e\right)$; if $\varphi_{r2}\leq \varphi_{r}<50\%$, then $\Pi_{m}^{\text{sr}}\left(w,e\right)\leq \Pi_{m}^{\text{nr}}\left(w,e\right)$; (b) when $\frac{1}{2}\leq u_{e}<\frac{9}{4}$, then $\Pi_{m}^{\text{sr}}\left(w,e\right)>\Pi_{m}^{\text{nr}}\left(w,e\right)$; and (ii) $\Pi_{r}^{\text{sr}}\left(p\right)>\Pi_{r}^{\text{nr}}\left(p\right)$, where $\varphi_{r2}=u_{e}$.

**Fig. 3:**
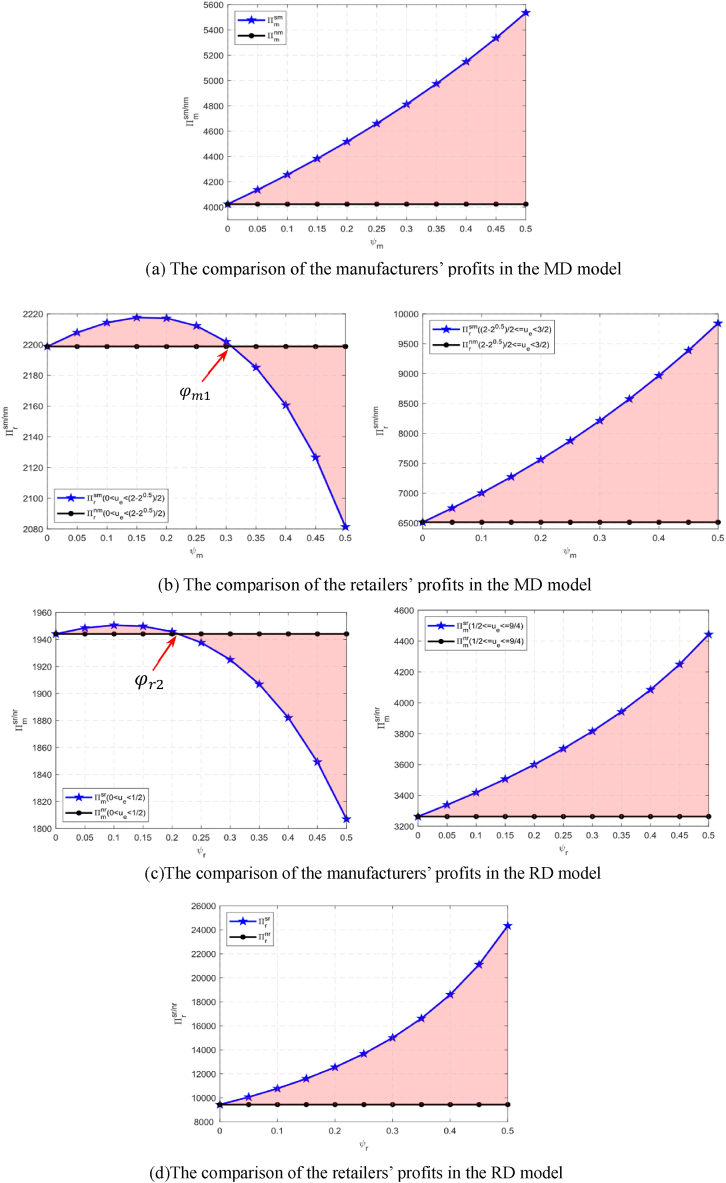
(1). The manufacturer’s preference and the retailer’s preference for the profits in the MD model. (a) The comparison of the manufacturers’ profits in the MD model (b) The comparison of the retailers’ profits in the MD model Fig. 3(2). The manufacturer’s preference and the retailer’s preference for the profits in the RD model. (d)The comparison of the retailers’ profits in the RD model (c)The comparison of the manufacturers’ profits in the RD model

Proposition 5 shows that leaders in the shareholding supply chains are always the beneficiaries (see Fig. 3(1) (a) and Fig. 3(2) (d)). However, the situations followers face are relatively complicated. In the MD model, when $0<u_{e}<\frac{2-\sqrt{2}}{2}$, there is a critical value $\varphi_{m1}$ for $\varphi_{m}$. If $\varphi_{m}$ is less than (greater than or equal to) $\varphi_{m1}$, the retailer’s profit in the shareholding model is greater than (less than or equal to) that of the retailer in the non-shareholding model. When $\frac{2-\sqrt{2}}{2}\leq u_{e}<\frac{3}{2}$, the profit of the retailer in the shareholding model is always greater than that in the non-shareholding model (see Fig. 3(1) (b)). In the RD model, when $0<u_{e}<\frac{1}{2}$, if $\varphi_{r}$ is less than (greater than or equal to) $\varphi_{r2}$, the profit of the manufacturer in the shareholding model is greater than (less than or equal to) than that in the non-shareholding model. When $\frac{1}{2}\leq u_{e}<\frac{9}{4}$, the manufacturer’s profit in the shareholding model is always greater than that in the non-shareholding model (see Fig. 3(2) (c)).

We find that for both the MD model and the RD model, when the market environment is bad, the follower’s profit in the shareholding model is still greater than that in the non-shareholding model as long as the share ratio of the leader to the follower is below a certain threshold. However, when the market environment is good, even if the followers’ profits are divided by the supply chain leaders, the followers’ profits are still greater than those in the non-shareholding model. Therefore, if the chain leaders decide to adopt a shareholding strategy for the followers, the followers can adjust the shareholding ratio according to the specific situation for their self-benefit.

## 5 Effects of technology spillovers

In practice, some firms voluntarily increase technology spillovers strategically. Therefore, in this section, we explore the impacts of the manufacturers’ spillovers on decisions and profits in each power structure. Appendix C provides the proofs of this section.

### 5.1 The effects of technology spillovers on decisions

Proposition 6 (1) $\frac{\partial e^{\text{ni}}}{\partial \theta }>0$, $\frac{\partial w^{\text{ni}}}{\partial \theta }>0$, $\frac{\partial p^{\text{ni}}}{\partial \theta }>0$ and $\frac{\partial q^{\text{ni}}}{\partial \theta }>0$, where i=m,r.

(2) $\frac{\partial e^{\text{si}}}{\partial \theta }>0$, $\frac{\partial w^{\text{si}}}{\partial \theta }>0$, $\frac{\partial p^{\text{si}}}{\partial \theta }>0$ and $\frac{\partial q^{\text{si}}}{\partial \theta }>0$, where i=m,r.

Part (1) of Proposition 6 shows that in two power structures, for the non-shareholding models, increasing the manufacturer’s technology spillovers result in a higher carbon emissions reduction level, which, in turn, leads to higher prices (both wholesale and selling). Part (2) of Proposition 6 implies that the same conclusions can be obtained for the shareholding models. Therefore, the impact of manufacturers’ technology spillovers on carbon reduction, pricing, and ordering decisions is independent of the power structure and shareholding strategy. The result shows that increasing technology spillovers is highly beneficial for achieving carbon emissions reduction goals.

### 5.2 The effects of technology spillovers on profits

The influence of spillover on retailers and manufacturers’ profits is as follows.

Proposition 7 (1) $\frac{\partial \Pi_{m}^{\text{ni}}\left(w,e\right)}{\partial \theta }>0$ and $\frac{\partial \Pi_{r}^{\text{ni}}\left(p\right)}{\partial \theta }>0$, where i=m,r.

(2) $\frac{\partial \Pi_{m}^{\text{si}}\left(w,e\right)}{\partial \theta }>0$ and $\frac{\partial \Pi_{r}^{\text{si}}\left(p\right)}{\partial \theta }>0$, where i=m,r.

The conclusions of Proposition 7 can also be derived from the results of Proposition 6. If the manufacturer increases his technology spillovers, the carbon emissions reduction of products will be improved, which helps attract more consumers. When prices increase, the profits of the individual supply chain members and the entire supply chain will increase. Obviously, regardless of the power structure and shareholding strategy, improving the manufacturer’s technology spillovers has positive effects.

### 5.3 The impact of supply chain leaders' shareholding strategies on manufacturers’ technology spillovers

In this subsection, we explore ways to stimulate manufacturers to increase their technology spillovers through the supply chain leader's shareholding strategy.

Proposition 8 (1) In the MD model, $\frac{\partial \theta }{\partial \varphi_{m}}>0$.

(2) In the RD model, (i) when $0<u_{e}<\frac{3}{4}$, if ${0<\varphi }_{r}<\varphi_{r3}$, then $\frac{\partial \theta }{\partial \varphi_{r}}>0$; if ${\varphi_{r3}\leq \varphi }_{r}<50\%$, then $\frac{\partial \theta }{\partial \varphi_{r}}\leq 0$; (ii) when $\frac{3}{4}\leq u_{e}<\frac{9}{4}$, then $\frac{\partial \theta }{\partial \varphi_{r}}>0$, where $\varphi_{r3}=1-\sqrt{1-u_{e}}$.

Proposition 8 shows that, in the MD model, shareholding has a positive impact on the manufacturer’s technology spillovers because the manufacturer’s profit will increase with the increase in holding retailer’s shares. In the RD model, when $0<u_{e}<\frac{3}{4}$, if the manufacturer’s shareholding ratio $\varphi_{r}$ is less than (greater than) the critical value $\varphi_{r3}$, the manufacturer’s technology spillover rate θ will increase (decrease) with the increase in $\varphi_{r}$. When $\frac{3}{4}\leq u_{e}<\frac{9}{4}$, the manufacturer’s technology spillover rate θ increases with $\varphi_{r}$. Therefore, the leader in the RD model should determine the shareholding ratio according to different market conditions to promote the manufacturer to improve the technology spillover level.

## 6 Supply chain coordination

A series of studies has covered the issue of supply chain coordination [59,60]. In this section, we discuss how to use a two-part tariff contract to achieve the coordination of the green shareholding supply chain under different power structures, which is a different approach from previous studies. The proofs of this section are provided in Appendix D**.**

### 6.1 Centralized supply chain model

The ideal state of supply chain coordination is that the manufacturer and the retailer make decisions with the goal of maximizing the integrated interests of the supply chain. The centralized supply chain model is:

(7) $$\Pi_{t}^{I}\left(e,p\right)=\left(p-c\right)\left[D-\varepsilon p+\lambda \left(1+\theta \right)e\right]-\frac{1}{2}z\left(1+\theta \right)e^{2}$$

Centralized decision making is aimed at maximizing the integrated profit of the supply chain, but the internal logic is still to make the carbon emissions reduction decision in the first stage and then the pricing decisions in the second stage. Based on Equation (7), the optimal solution of the centralized supply chain model is shown in the following lemma.

Lemma 1 $e^{I}=\frac{\left(D-\varepsilon c\right)\lambda }{2\varepsilon z-\lambda^{2}\left(1+\theta \right)}$, $p^{I}=c+\frac{z\left(D-\varepsilon c\right)}{2\varepsilon z-\lambda^{2}\left(1+\theta \right)}$, $q^{I}=\frac{\varepsilon z\left(D-\varepsilon c\right)}{2\varepsilon z-\lambda^{2}\left(1+\theta \right)}$ and $\Pi_{t}^{I}\left(e,p\right)=\frac{z{\left(D-\varepsilon c\right)}^{2}}{2\left[2\varepsilon z-\lambda^{2}\left(1+\theta \right)\right]}$.

Lemma 1 shows that there is a unique optimal carbon emissions reduction level and product price for a supply chain with centralized decision making. Next, we compare the profits of decentralized and centralized decision supply chains.

Corollary 1 The profit of a centralized supply chain is greater than that of a shareholding supply chain, that is, $\Pi_{t}^{I}\left(e^{I},p^{I}\right)>\Pi_{t}^{\text{si}}\left(w^{\text{si}}{,e}^{\text{si}},p^{\text{si}}\right)$, where i=m,r.

**Fig. 4:**
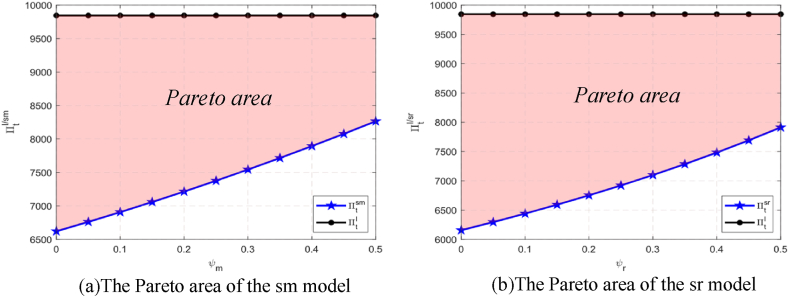
The Pareto areas of the supply chains. (b)The Pareto area of the sr model (a)The Pareto area of the sm model

Proposition 4 indicates that the shareholding strategy can improve the overall income of the supply chain. However, Corollary 1 shows that the profit of the centralized supply chain is still better than that of the shareholding supply chain, and Fig. 4(a and b) visualizes it. Nevertheless, for upstream and downstream firms in the supply chain, it is difficult to achieve centralized decision making, so we will apply the two-part tariff contract to coordinate the shareholding supply chain in the next section.

### 6.2 Two-part tariff contract model

We use the two-part tariff contract to coordinate the shareholding supply chains under different power structures. The manufacturer undertakes technology investment to reduce carbon emissions, and the retailer pays the manufacturer a lump-sum payment to coordinate the supply chain.

In the MD model, applying the two-part tariff contract to coordinate the shareholding supply chain (labelled with superscript MT), the manufacturer's profit function is

$$\Pi_{m}^{MT}\left(w,e\right)=\left(w-c\right)\left[D-\varepsilon p+\lambda \left(1+\theta \right)e\right]-\frac{1}{2}z\left(1+\theta \right)e^{2}$$

(8) $$+\varphi_{m}\left(p-w\right)\left[D-\varepsilon p+\lambda \left(1+\theta \right)e\right]+M^{sm}$$

The retailer's profit function is

(9) $$\Pi_{r}^{MT}\left(p\right)=\left(1-\varphi_{m}\right)\left(p-w\right)\left[D-\varepsilon p+\lambda \left(1+\theta \right)e\right]-M^{sm}$$

In the RD model, applying the two-part tariff contract to coordinate the supply chain (labelled with superscript RT), the manufacturer's profit function is

(10) $$\Pi_{m}^{RT}\left(w,e\right)=\left(1-\varphi_{r}\right)\left(w-c\right)\left[D-\varepsilon p+\lambda \left(1+\theta \right)e\right]-\left(1-\varphi_{r}\right)\frac{1}{2}z\left(1+\theta \right)e^{2}+M^{sr}$$

The retailer's profit function is

$$\Pi_{r}^{RT}\left(p\right)=\left(p-w\right)\left[D-\varepsilon p+\lambda \left(1+\theta \right)e\right]+\varphi_{r}\left(w-c\right)\left[D-\varepsilon p+\lambda \left(1+\theta \right)e\right]$$

(11) $$-\varphi_{r}\frac{1}{2}z\left(1+\theta \right)e^{2}-M^{\text{sr}}$$

Based on Equations (8), (9), (10), (11), we use the two-part tariff contract to coordinate the shareholding supply chain and obtain the following conclusions.

Proposition 9 Applying the two-part tariff contract to coordinate the shareholding supply chain satisfies $w^{\text{si}}=c,\left(i=m,r\right)$.

(1) In the MD model, $M^{\text{sm}}=\frac{4\varepsilon^{2}z^{3}{\left(1-\varphi_{m}\right)}^{2}{\left(D-\varepsilon c\right)}^{2}\left[\left(3-\varphi_{m}\right)\varepsilon z-\lambda^{2}\left(1+\theta \right)\right]}{{[2\varepsilon z-\lambda^{2}\left(1+\theta \right)]}^{2}{[2\varepsilon z\left(2-\varphi_{m}\right)-\lambda^{2}\left(1+\theta \right)]}^{2}}$.
(2) In the RD model, $M^{\text{sr}}=\frac{\varepsilon z^{2}\left(1-\varphi_{r}\right){\left(D-\varepsilon c\right)}^{2}\left[{\left(2-\varphi_{r}\right)}^{2}\lambda^{2}\left(1+\theta \right)+2\varepsilon z-2\lambda^{2}\left(1+\theta \right)\right]}{{[2\varepsilon z-\lambda^{2}\left(1+\theta \right)]}^{2}[2\varepsilon z{\left(2-\varphi_{r}\right)}^{2}-\lambda^{2}\left(1+\theta \right)]}$.

Proposition 9 shows that supply chain coordination can be achieved by applying the two-part tariff contract, in which both manufacturers and retailers will benefit, and their profits are greater than without contract coordination. The influence of the leader’s shareholding ratio $\varphi_{i}\left(i=m,r\right)$ on the lump-sum fee $M^{\text{si}}\left(i=m,r\right)$ paid by the retailer to the manufacturer is as follows.

Lemma 2 $\frac{\partial M^{\text{si}}}{\partial \varphi_{i}}<0$, where i=m,r.

Lemma 2 shows that the lump-sum fee $M^{\text{si}}$ paid by the retailer to the manufacturer is affected by the shareholding ratio $\varphi_{i}$. If the leader’s shareholding ratio $\varphi_{i}$ increases, the lump-sum fee $M^{\text{si}}$ will decrease in each power structure. Combined with Proposition 4, it can be seen that, if the shareholding ratio $\varphi_{i}$ increases, the profit of the decentralized supply chain will increase and the profit difference between the centralized supply chain and the decentralized supply chain will decrease. The result indicates that, when $\varphi_{i}$ increases, the Pareto improvement area of applying the two-part tariff contract to coordinate the shareholding supply chain decreases.

## 7 Conclusion

We investigate a green supply chain in which a leader holds equity in a follower under different power structures. Through comparisons with non-shareholding-models, we focus on analyzing the decision-making and profit-sharing mechanisms of the equity strategic alliance in green supply chains. Furthermore, we investigate the effect of a manufacturer's technology spillovers on the chain's environmental and economic performance and explore ways to increase the manufacturer's technology spillovers through shareholding strategies. Finally, we coordinate the green shareholding supply chains under a two-part tariff contract. The research results may provide decision-making references for promoting low-carbon economy, thereby improving supply chain efficiency and government regulation.

Based on this paper's results, we can draw the following management implications.

(1) The application of vertical shareholding strategies by core firms in green supply chains can help reduce carbon emissions and improve the overall economic efficiency of the supply chain. Therefore, the government can implement policies that foster shareholding cooperation among green supply chain firms to achieve a carbon-efficient economy. In China, the government has established comprehensive guidelines for state-owned enterprises to engage in equity participation in business and investment operations.
(2) Regardless of the market environment, leaders of green supply chains can increase profits by increasing their shareholdings of followers. Therefore, from the perspective of the supply chain leader, the leader should increase their shareholding ratio of the follower. For example, as a leader in the e-commerce supply chain, Alibaba holds a 25.25 % stake in Haier.
(3) The follower's profit has different trends in different market environments and is subject to the shareholding ratio. Therefore, from the perspective of the supply chain follower, the follower can only benefit by accepting the appropriate shareholding ratio according to different market environments. Furthermore, supply chain leaders can determine the appropriate shareholding ratio according to different market environments to achieve a win-win situation with followers, thus reducing resistance to the implementation of the shareholding strategy.
(4) Independent of power position, increased technology spillovers from manufacturers benefit not only themselves but also downstream retailers. Hence, governments should implement reasonable measures to protect intellectual property and encourage the exchange of relevant technologies within green supply chains. For instance, the Chinese government established the China Technology Exchange with the aim of protecting intellectual property and promoting technology exchanges.
(5) The two-part tariff contract can achieve effective coordination between upstream and downstream members of the supply chain when it is difficult for them to achieve centralized decision-making.

This paper examines the impact of core firms’ shareholding strategies on the management and operation of green supply chains in terms of the economy and the environment. In the future, the research results can be further enriched in the following directions. First, investment institutions play an important role in equity investment, and their influence on supply chain decisions cannot be ignored. Second, to achieve carbon peaking and carbon neutrality goals, firms are faced with the constraints of carbon cap policies, and it is necessary to explore the impact of various environmental protection policies on the shareholding supply chain. Finally, with the launch of the carbon trading market, supply chain firms have new financing channels; thus, it would be useful to explore the impact of carbon finance on the supply chain of shareholding.

## Funding statement

This study was funded by the National Natural Science Foundation, China (No. 71873111; 71273214), and the Humanities and Social Sciences Fund by the Ministry of Education, China (No.18YJAZH024).

## Data availability statement

No data was used for the research described in the article.

## CRediT authorship contribution statement

**Wenqiang Li:** Writing – review & editing, Writing – original draft, Software, Methodology, Conceptualization. **Juan He:** Investigation, Funding acquisition. **Yangyan Shi:** Validation, Methodology.

## Declaration of competing interest

The authors declare that they have no known competing financial interests or personal relationships that could have appeared to influence the work reported in this paper.

## Appendix A

### The derivation of Table 3

(1) In the nm model (i=m), from $\Pi_{r}^{nm}\left(p\right)$, we obtain $\frac{\partial^{2}\Pi_{r}^{nm}\left(p\right)}{\partial p^{2}}=-2\varepsilon <0$, so $\Pi_{r}^{nm}\left(p\right)$ is a concave function of p. Let $\frac{\partial \Pi_{r}^{nm}\left(p\right)}{\partial p}=0$, and we obtain $p\left(w,e\right)=\frac{D+\varepsilon w+\lambda \left(1+\theta \right)e}{2\varepsilon }$; we replace p(w,e) in $\Pi_{m}^{nm}\left(w,e\right)$, and we obtain $\frac{\partial^{2}\Pi_{m}^{nm}\left(w,e\right)}{\partial w^{2}}=-\varepsilon <0$. Let $\frac{\partial \Pi_{m}^{nm}\left(w,e\right)}{\partial w}=0$, and we obtain $w\left(e\right)=\frac{D+\varepsilon c+\lambda \left(1+\theta \right)e}{2\varepsilon }$. We replace w(e) in p(w,e), and we obtain $p\left(e\right)=\frac{3D+\varepsilon c+3\lambda \left(1+\theta \right)e}{4\varepsilon }$, and then we replace w(e) and p(e) in $\Pi_{m}^{nm}\left(w,e\right)$ and obtain $\frac{\partial^{2}\Pi_{m}^{nm}\left(w,e\right)}{\partial e^{2}}=\frac{\lambda^{2}{\left(1+\theta \right)}^{2}}{4\varepsilon }-z\left(1+\theta \right)$. To ensure that the model has a solution, we assume $\frac{\lambda^{2}{\left(1+\theta \right)}^{2}}{4\varepsilon }-z\left(1+\theta \right)<0$, that is, $\frac{\lambda^{2}\left(1+\theta \right)}{2\varepsilon z}<2$; let $u_{e}=\frac{\lambda^{2}\left(1+\theta \right)}{2\varepsilon z}$, then $0<u_{e}<2$. Let $\frac{\partial \Pi_{m}^{nm}\left(w,e\right)}{\partial e}=0$, and we obtain $e^{nm}=\frac{\left(D-\varepsilon c\right)\lambda }{4\varepsilon z-\lambda^{2}\left(1+\theta \right)}$ and replace $e^{nm}$ in w(e) and p(e), respectively. We obtain $w^{nm}=c+\frac{2z\left(D-\varepsilon c\right)}{4\varepsilon z-\lambda^{2}\left(1+\theta \right)}$, $p^{nm}=c+\frac{3z\left(D-\varepsilon c\right)}{4\varepsilon z-\lambda^{2}\left(1+\theta \right)}$. By calculation, we can obtain the following results: $q^{nm}=\frac{\varepsilon z\left(D-\varepsilon c\right)}{4\varepsilon z-\lambda^{2}\left(1+\theta \right)}$, $\Pi_{m}^{nm}\left(w,e\right)=\frac{z{\left(D-\varepsilon c\right)}^{2}}{2\left[4\varepsilon z-\lambda^{2}\left(1+\theta \right)\right]}$ and $\Pi_{r}^{nm}\left(p\right)=\frac{\varepsilon z^{2}{\left(D-\varepsilon c\right)}^{2}}{{[4\varepsilon z-\lambda^{2}\left(1+\theta \right)]}^{2}}$.
(2) In the nr model (i=r), from $\Pi_{m}^{nr}\left(w,e\right)$, we obtain $\frac{\partial^{2}\Pi_{m}^{nr}\left(w,e\right)}{\partial w^{2}}=-2\varepsilon <0$, so $\Pi_{m}^{nr}\left(w,e\right)$ is a concave function of w. Let $\frac{\partial \Pi_{m}^{nr}\left(w,e\right)}{\partial w}=0$, and we obtain $w\left(p,e\right)=\frac{D-\varepsilon p+\lambda \left(1+\theta \right)e+\varepsilon c}{2\varepsilon }$. By replacing w(p,e) in $\Pi_{r}^{nr}\left(p\right)$, we obtain $\frac{\partial^{2}\Pi_{r}^{nr}\left(p\right)}{\partial p^{2}}=-4\varepsilon <0$. Let $\frac{\partial \Pi_{r}^{nr}\left(p\right)}{\partial p}=0$, and we obtain $p\left(e\right)=\frac{3D+\varepsilon c+3\lambda \left(1+\theta \right)e}{4\varepsilon }$. We replace w(p,e) and p(e) in $\Pi_{m}^{nr}\left(w,e\right)$, then we have $\frac{\partial^{2}\Pi_{m}^{nr}\left(w,e\right)}{\partial e^{2}}=\frac{\lambda^{2}{\left(1+\theta \right)}^{2}}{8\varepsilon }-z\left(1+\theta \right)$. To ensure that the model has a solution, we assume $\frac{\lambda^{2}{\left(1+\theta \right)}^{2}}{8\varepsilon }-z\left(1+\theta \right)<0$, then we obtain $0<u_{e}<4$. Let $\frac{\partial \Pi_{m}^{nr}\left(w,e\right)}{\partial e}=0$, and we obtain $e^{nr}=\frac{\left(D-\varepsilon c\right)\lambda }{8\varepsilon z-\lambda^{2}\left(1+\theta \right)}$. Substitute $e^{nr}$ into p(e) to get $p^{nr}=c+\frac{6z\left(D-\varepsilon c\right)}{8\varepsilon z-\lambda^{2}\left(1+\theta \right)}$. By Replacing $e^{nr}$ and $p^{nr}$ in w(p,e), we obtain $w^{nr}=c+\frac{2z\left(D-\varepsilon c\right)}{8\varepsilon z-\lambda^{2}\left(1+\theta \right)}$. In addition, we can get the following results: $q^{nr}=\frac{2\varepsilon z\left(D-\varepsilon c\right)}{8\varepsilon z-\lambda^{2}\left(1+\theta \right)}$, $\Pi_{m}^{nr}\left(w,e\right)=\frac{z{\left(D-\varepsilon c\right)}^{2}}{2\left[8\varepsilon z-\lambda^{2}\left(1+\theta \right)\right]}$, $\Pi_{r}^{nr}\left(p\right)=\frac{8\varepsilon z^{2}{\left(D-\varepsilon c\right)}^{2}}{{\left[8\varepsilon z-\lambda^{2}\left(1+\theta \right)\right]}^{2}}$.
(3) In the sm model (i=m), from $\Pi_{r}^{sm}\left(p\right)$, we obtain $\frac{\partial^{2}\Pi_{r}^{sm}\left(p\right)}{\partial p^{2}}=-2\varepsilon \left(1-\varphi_{m}\right)<0$, so $\Pi_{r}^{sm}\left(p\right)$ is a concave function of p. Let $\frac{\partial \Pi_{r}^{sm}\left(p\right)}{\partial p}=0$, and we obtain $p\left(w,e\right)=\frac{D+\varepsilon w+\lambda \left(1+\theta \right)e}{2\varepsilon }$. We replace p(w,e) in $\Pi_{m}^{sm}\left(w,e\right)$ and get $\frac{\partial^{2}\Pi_{m}^{sm}\left(w,e\right)}{\partial w^{2}}=-\frac{\varepsilon \left(2-\varphi_{m}\right)}{2}<0$. Let $\frac{\partial \Pi_{m}^{sm}\left(w,e\right)}{\partial w}=0$, and we obtain $w\left(e\right)=\frac{\left(1-\varphi_{m}\right)D+\varepsilon c+\left(1-\varphi_{m}\right)\lambda \left(1+\theta \right)e}{\left(2-\varphi_{m}\right)\varepsilon }$. Replace w(e) in p(w,e), and we get $p\left(e\right)=\frac{\left(3-2\varphi_{m}\right)D+\varepsilon c+\left(3-2\varphi_{m}\right)\lambda \left(1+\theta \right)e}{2\varepsilon \left(2-\varphi_{m}\right)}$. Substitute w(e) and p(e) into $\Pi_{m}^{sm}\left(w,e\right)$ to get $\frac{\partial^{2}\Pi_{m}^{sm}\left(w,e\right)}{\partial e^{2}}=\frac{\lambda^{2}{\left(1+\theta \right)}^{2}}{2\varepsilon \left(2-\varphi_{m}\right)}-z\left(1+\theta \right)$. To ensure that the model has a solution, we assume $\frac{\lambda^{2}{\left(1+\theta \right)}^{2}}{2\varepsilon \left(2-\varphi_{m}\right)}-z\left(1+\theta \right)<0$, that is $\frac{\lambda^{2}\left(1+\theta \right)}{2\varepsilon z}<\min\;\left(2-\varphi_{m}\right)$, because $0\leq \varphi_{m}\leq 50\%$, so $0<u_{e}<\frac{3}{2}$. Let $\frac{\partial \Pi_{m}^{sm}\left(w,e\right)}{\partial e}=0$, and we get $e^{sm}=\frac{\left(D-\varepsilon c\right)\lambda }{2\varepsilon z\left(2-\varphi_{m}\right)-\lambda^{2}\left(1+\theta \right)}$. Replace $e^{sm}$ in w(e) and p(e), respectively. We obtain $w^{sm}=c+\frac{2z\left(1-\varphi_{m})(D-\varepsilon c\right)}{2\varepsilon z\left(2-\varphi_{m}\right)-\lambda^{2}\left(1+\theta \right)}$, $p^{sm}=c+\frac{z\left(3-{2\varphi }_{m})(D-\varepsilon c\right)}{2\varepsilon z\left(2-\varphi_{m}\right)-\lambda^{2}\left(1+\theta \right)}$. In addition, we can get the following results: $q^{sm}=\frac{\varepsilon z\left(D-\varepsilon c\right)}{2\varepsilon z\left(2-\varphi_{m}\right)-\lambda^{2}\left(1+\theta \right)}$, $\Pi_{m}^{sm}\left(w,e\right)=\frac{z{\left(D-\varepsilon c\right)}^{2}}{2\left[2\varepsilon z\left(2-\varphi_{m}\right)-\lambda^{2}\left(1+\theta \right)\right]}$, $\Pi_{r}^{sm}\left(p\right)=\frac{\left(1-\varphi_{m}\right)\varepsilon z^{2}{\left(D-\varepsilon c\right)}^{2}}{{\left[2\varepsilon z\left(2-\varphi_{m}\right)-\lambda^{2}\left(1+\theta \right)\right]}^{2}}$.
(4) In the sr model (i=r), from $\Pi_{m}^{sr}\left(w,e\right)$, we obtain $\frac{\partial^{2}\Pi_{m}^{sr}\left(w,e\right)}{\partial w^{2}}=-2\varepsilon \left(1-\varphi_{r}\right)<0$, so $\Pi_{m}^{sr}\left(w,e\right)$ is a concave function of w. Let $\frac{\partial \Pi_{m}^{sr}\left(w,e\right)}{\partial w}=0$, and we obtain $w\left(p,e\right)=\frac{D-\varepsilon p+\lambda \left(1+\theta \right)e+\varepsilon c}{\varepsilon }$. Substitute w(p,e) into $\Pi_{r}^{sr}\left(p\right)$ to get $\frac{\partial^{2}\Pi_{r}^{sr}\left(p\right)}{\partial p^{2}}=-2\varepsilon \left(2-\varphi_{r}\right)<0$. Let $\frac{\partial \Pi_{r}^{sr}\left(p\right)}{\partial p}=0$, and we obtain $p\left(e\right)=\frac{\left(3-{2\varphi }_{r}\right)D+\varepsilon c+\left(3-{2\varphi }_{r}\right)\lambda \left(1+\theta \right)e}{2\left(2-\varphi_{r}\right)\varepsilon }$. By Replacing w(p,e) and p(e) in $\Pi_{m}^{sr}\left(w,e\right)$, we obtain $\frac{\partial^{2}\Pi_{m}^{sr}\left(w,e\right)}{\partial e^{2}}=\left(1-\varphi_{r}\right)\left[\frac{\lambda^{2}{\left(1+\theta \right)}^{2}}{2\varepsilon {\left(2-\varphi_{r}\right)}^{2}}-z\left(1+\theta \right)\right]$. To ensure that the model has a solution, we assume $\frac{\lambda^{2}{\left(1+\theta \right)}^{2}}{2\varepsilon {\left(2-\varphi_{r}\right)}^{2}}-z\left(1+\theta \right)<0$, that is $\frac{\lambda^{2}\left(1+\theta \right)}{2\varepsilon z}<\min\;{\left(2-\varphi_{r}\right)}^{2}$, because $0\leq \varphi_{r}\leq 50\%$, so $0<u_{e}<\frac{9}{4}$. Let $\frac{\partial \Pi_{m}^{sr}\left(w,e\right)}{\partial e}=0$, and we get $e^{sr}=\frac{\left(D-\varepsilon c\right)\lambda }{2\varepsilon z{\left(2-\varphi_{r}\right)}^{2}-\lambda^{2}\left(1+\theta \right)}$. Substitute $e^{sr}$ into p(e) to get $p^{sr}=c+\frac{z\left(D-\varepsilon c\right)\left(3-{2\varphi }_{r}\right)\left(2-\varphi_{r}\right)}{2\varepsilon z{\left(2-\varphi_{r}\right)}^{2}-\lambda^{2}\left(1+\theta \right)}$. Then we replace $e^{sr}$ and $p^{sr}$ in w(p,e) and obtain $w^{sr}=c+\frac{\left(2-\varphi_{r})z(D-\varepsilon c\right)}{2\varepsilon z{\left(2-\varphi_{r}\right)}^{2}-\lambda^{2}\left(1+\theta \right)}$. In addition, we can get the following results: $q^{sr}=\frac{\varepsilon z\left(D-\varepsilon c\right)\left(2-\varphi_{r}\right)}{2\varepsilon z{\left(2-\varphi_{r}\right)}^{2}-\lambda^{2}\left(1+\theta \right)}$, $\Pi_{m}^{sr}\left(w,e\right)=\frac{\left(1-\varphi_{r}\right)z{\left(D-\varepsilon c\right)}^{2}}{2\left[2\varepsilon z{\left(2-\varphi_{r}\right)}^{2}-\lambda^{2}\left(1+\theta \right)\right]}$,

$$\Pi_{r}^{sr}\left(p\right)=\frac{z{\left(D-\varepsilon c\right)}^{2}\left[2\varepsilon z{\left(2-\varphi_{r}\right)}^{3}-\varphi_{r}\lambda^{2}\left(1+\theta \right)\right]}{{2\left[2\varepsilon z{\left(2-\varphi_{r}\right)}^{2}-\lambda^{2}\left(1+\theta \right)\right]}^{2}}$$

## Appendix B

Proof of Proposition 1.

(1) Given $0<\varphi_{m}<50\%$, (i) $e^{sm}-e^{nm}=\frac{2\varepsilon z\varphi_{m}\left(D-\varepsilon c\right)\lambda }{\left[2\varepsilon z\left(2-\varphi_{m}\right)-\lambda^{2}\left(1+\theta \right)\right]\left[4\varepsilon z-\lambda^{2}\left(1+\theta \right)\right]}>0$.

(ii) $q^{sm}-q^{nm}=\frac{2\varepsilon^{2}z^{2}\varphi_{m}\left(D-\varepsilon c\right)}{\left[2\varepsilon z\left(2-\varphi_{m}\right)-\lambda^{2}\left(1+\theta \right)\right]\left[4\varepsilon z-\lambda^{2}\left(1+\theta \right)\right]}>0$.

(2) Given $0<\varphi_{r}<50\%$, (i) $e^{sr}-e^{nr}=\frac{2\varepsilon z\left(D-\varepsilon c\right)\lambda \left[4-{\left(2-\varphi_{r}\right)}^{2}\right]}{\left[2\varepsilon z{\left(2-\varphi_{r}\right)}^{2}-\lambda^{2}\left(1+\theta \right)\right]\left[8\varepsilon z-\lambda^{2}\left(1+\theta \right)\right]}>0$.

(ii) $q^{sr}-q^{nr}=\frac{\varepsilon z\left(D-\varepsilon c\right)\varphi_{r}\left[4\varepsilon z\left(2-\varphi_{r}\right)+\lambda^{2}\left(1+\theta \right)\right]}{\left[2\varepsilon z{\left(2-\varphi_{r}\right)}^{2}-\lambda^{2}\left(1+\theta \right)\right]\left[8\varepsilon z-\lambda^{2}\left(1+\theta \right)\right]}>0$.

Proof of Proposition 2.

(1) Given $0<\varphi_{m}<50\%$, from $w^{sm}-w^{nm}=\frac{2z\varphi_{m}\left(D-\varepsilon c\right)\left[\lambda^{2}\left(1+\theta \right)-2\varepsilon z\right]}{\left[2\varepsilon z\left(2-\varphi_{m}\right)-\lambda^{2}\left(1+\theta \right)\right]\left[4\varepsilon z-\lambda^{2}\left(1+\theta \right)\right]}$, we know that the size relationship between $w^{sm}$ and $w^{nm}$ is determined by $\lambda^{2}\left(1+\theta \right)-2\varepsilon z$. Therefore, we obtain (a) when $0<u_{e}<1$, then $w^{sm}<w^{nm}$. (b) when $u_{e}=1$, then $w^{sm}=w^{nm}$. (c) when $1<u_{e}<\frac{3}{2}$, then $w^{sm}>w^{nm}$.
(2) Given ${0<\varphi }_{r}<50\%$, $w^{sr}-w^{nr}=\frac{z\left(D-\varepsilon c\right)\varphi_{r}\left[4\varepsilon z\left(2-\varphi_{r}\right)+\lambda^{2}\left(1+\theta \right)\right]}{\left[2\varepsilon z{\left(2-\varphi_{r}\right)}^{2}-\lambda^{2}\left(1+\theta \right)\right]\left[8\varepsilon z-\lambda^{2}\left(1+\theta \right)\right]}>0$.

Proof of Proposition 3.

(2) Given ${0<\varphi }_{r}<50\%$, from $p^{sr}-p^{nr}=\frac{z\left(D-\varepsilon c\right)\varphi_{r}\left[{\left(7-2\varphi_{r}\right)\lambda }^{2}\left(1+\theta \right)-4\varepsilon z\left(2-\varphi_{r}\right)\right]}{\left[2\varepsilon z{\left(2-\varphi_{r}\right)}^{2}-\lambda^{2}\left(1+\theta \right)\right]\left[8\varepsilon z-\lambda^{2}\left(1+\theta \right)\right]}$, we know that the size relationship between $p^{sr}$ and $p^{nr}$ is determined by ${\left(7-2\varphi_{r}\right)\lambda }^{2}\left(1+\theta \right)-4\varepsilon z\left(2-\varphi_{r}\right)$. Making $f_{1}\left(\varphi_{r}\right)=\frac{2\left(2-\varphi_{r}\right)}{7-2\varphi_{r}}$, we obtain $\frac{\partial f_{1}\left(\varphi_{r}\right)}{\partial \varphi_{r}}=-\frac{6}{{\left(7-2\varphi_{r}\right)}^{2}}<0$. Therefore, when $0<\varphi_{r}<50\%$, then ${\frac{1}{2}<f}_{1}\left(\varphi_{r}\right)<\frac{4}{7}$. Inter-partition discussion: (a) when $0<u_{e}\leq \frac{1}{2}$, then $p^{sr}<p^{nr}$. (b) when $\frac{1}{2}<u_{e}<\frac{4}{7}$, making $f_{2}\left(\varphi_{r}\right)=2\left(u_{e}-1\right)\varphi_{r}+4-7u_{e}$ and letting $f_{2}\left(\varphi_{r}\right)=0$, we obtain $\varphi_{r1}=\frac{4-7u_{e}}{2\left(1-u_{e}\right)}$. From $\frac{\partial \varphi_{r1}}{\partial u_{e}}=-\frac{3}{2{\left(1-u_{e}\right)}^{2}}<0$, we obtain ${0<\varphi }_{r1}<50\%$. Further, if $0<\varphi_{r}<\varphi_{r1}$, then $p^{sr}<p^{nr}$; if $\varphi_{r1}\leq \varphi_{r}<50\%$, then $p^{sr}\geq p^{nr}$. (c) when $\frac{4}{7}\leq u_{e}<\frac{9}{4}$, then $p^{sr}>p^{nr}$.

Proof of Proposition 4.

(1) Given $0<\varphi_{m}<50\%$, $\frac{\partial \Pi_{t}^{sm}\left(w,e,p\right)}{\partial \varphi_{m}}=\frac{4\varepsilon^{2}z^{3}{\left(D-\varepsilon c\right)}^{2}\left(1-\varphi_{m}\right)}{{\left[2\varepsilon z\left(2-\varphi_{m}\right)-\lambda^{2}\left(1+\theta \right)\right]}^{3}}>0$. When $\varphi_{m}=0$, we have $\Pi_{t}^{sm}\left(w,e,p\right)=\Pi_{t}^{nm}\left(w,e,p\right)$; when $0<\varphi_{m}<50\%$, there must be $\Pi_{t}^{sm}\left(w,e,p\right)>\Pi_{t}^{nm}\left(w,e,p\right)$.
(2) Given ${0<\varphi }_{r}<50\%$,

$\frac{\partial \Pi_{t}^{sr}\left(w,e,p\right)}{\partial \varphi_{r}}=\frac{2\varepsilon z^{2}{\left(D-\varepsilon c\right)}^{2}\left(2-\varphi_{r}\right)\left(1-\varphi_{r}\right)\left[2\varepsilon z{\left(2-\varphi_{r}\right)}^{2}+3\lambda^{2}\left(1+\theta \right)\right]}{{\left[2\varepsilon z{\left(2-\varphi_{r}\right)}^{2}-\lambda^{2}\left(1+\theta \right)\right]}^{3}}>0$. When $\varphi_{r}=0$, we have $\Pi_{t}^{sr}\left(w,e,p\right)=\Pi_{t}^{nr}\left(w,e,p\right)$; when $0<\varphi_{r}<50\%$, there must be $\Pi_{t}^{sr}\left(w,e,p\right)>\Pi_{t}^{nr}\left(w,e,p\right)$.

Proof of Proposition 5.

(1) Given $0<\varphi_{m}<50\%$,
  (i) $\Pi_{m}^{sm}\left(w,e\right)-\Pi_{m}^{nm}\left(w,e\right)=\frac{\varepsilon z^{2}\varphi_{m}{\left(D-\varepsilon c\right)}^{2}}{\left[2\varepsilon z\left(2-\varphi_{m}\right)-\lambda^{2}\left(1+\theta \right)\right]\left[4\varepsilon z-\lambda^{2}\left(1+\theta \right)\right]}>0$.
  (ii) from $\Pi_{r}^{sm}\left(p\right)-\Pi_{r}^{nm}\left(p\right)=\frac{\varepsilon z^{2}\varphi_{m}{\left(D-\varepsilon c\right)}^{2}\left[4\varepsilon z\lambda^{2}\left(1+\theta \right)-4\varepsilon^{2}z^{2}\varphi_{m}-\lambda^{4}{\left(1+\theta \right)}^{2}\right]}{{\left[2\varepsilon z\left(2-\varphi_{m}\right)-\lambda^{2}\left(1+\theta \right)\right]}^{2}{\left[4\varepsilon z-\lambda^{2}\left(1+\theta \right)\right]}^{2}}$, we know that the size relationship between $\Pi_{r}^{sm}\left(p\right)$ and $\Pi_{r}^{nm}\left(p\right)$ is determined by $4\varepsilon z\lambda^{2}\left(1+\theta \right)-4\varepsilon^{2}z^{2}\varphi_{m}-\lambda^{4}{\left(1+\theta \right)}^{2}$. When $4\varepsilon z\lambda^{2}\left(1+\theta \right)-4\varepsilon^{2}z^{2}\varphi_{m}-\lambda^{4}{\left(1+\theta \right)}^{2}$ is greater than (less than or equal to) 0, $1-\sqrt{1-\varphi_{m}}$ is less than (more than or equal to) $u_{e}$. Making $f\left(\varphi_{m}\right)=1-\sqrt{1-\varphi_{m}}$, we get $\frac{\partial f\left(\varphi_{m}\right)}{\partial \varphi_{m}}=\frac{1}{2\sqrt{1-\varphi_{m}}}>0$, so $0<f\left(\varphi_{m}\right)<\frac{2-\sqrt{2}}{2}$. Inter-partition discussion: (a) when $0<u_{e}<\frac{2-\sqrt{2}}{2}$, if $0<\varphi_{m}<1-{\left(1-u_{e}\right)}^{2}$, then $\Pi_{r}^{sm}\left(p\right)>\Pi_{r}^{nm}\left(p\right)$; if $1-{\left(1-u_{e}\right)}^{2}\leq \varphi_{m}<50\%$, then $\Pi_{r}^{sm}\left(p\right)\leq \Pi_{r}^{nm}\left(p\right)$. (b) when $\frac{2-\sqrt{2}}{2}\leq u_{e}<\frac{3}{2}$, then $\Pi_{r}^{sm}\left(p\right)>\Pi_{r}^{nm}\left(p\right)$. For the convenience of expression, we denote that $\varphi_{m1}$ is $1-{\left(1-u_{e}\right)}^{2}$.
(2) Given $0<\varphi_{r}<50\%$,
  (i) from $\Pi_{m}^{sr}\left(w,e\right)-\Pi_{m}^{nr}\left(w,e\right)=\frac{z{\left(D-\varepsilon c\right)}^{2}\varphi_{r}\left[\lambda^{2}\left(1+\theta \right)-2\varepsilon z\varphi_{r}\right]}{2\left[2\varepsilon z{\left(2-\varphi_{r}\right)}^{2}-\lambda^{2}\left(1+\theta \right)\right]\left[8\varepsilon z-\lambda^{2}\left(1+\theta \right)\right]}$, we know that the size relationship between $\Pi_{m}^{sr}\left(w,e\right)$ and $\Pi_{m}^{nr}\left(w,e\right)$ is determined by $\lambda^{2}\left(1+\theta \right)-2\varepsilon z\varphi_{r}$. Inter-partition discussion: (a) when $0<u_{e}<\frac{1}{2}$, if $0<\varphi_{r}<u_{e}$, then $\Pi_{m}^{sr}\left(w,e\right)>\Pi_{m}^{nr}\left(w,e\right)$; if $u_{e}\leq \varphi_{r}<50\%$, then $\Pi_{m}^{sr}\left(w,e\right)\leq \Pi_{m}^{nr}\left(w,e\right)$. (b) when $\frac{1}{2}\leq u_{e}<\frac{9}{4}$, then $\Pi_{m}^{sr}\left(w,e\right)>\Pi_{m}^{nr}\left(w,e\right)$. (ii) when $\varphi_{r}=0$, there is $\Pi_{r}^{sr}\left(p\right)=\Pi_{r}^{nr}\left(p\right)$; For $\frac{{\partial \Pi }_{r}^{sr}\left(p\right)}{\partial \varphi_{r}}>0$, when ${0<\varphi }_{r}<50\%$, there must be $\Pi_{r}^{sr}\left(p\right)>\Pi_{r}^{nr}\left(p\right)$. For the convenience of expression, we denote that $\varphi_{r2}$ is $u_{e}$.

## Appendix C

Proof of Proposition 6.

(1) $\frac{\partial e^{nm}}{\partial \theta }=\frac{\lambda \left(D-\varepsilon c\right)[4\varepsilon z+\lambda^{2}\left(1+\theta \right)]}{{\left(1+\theta \right)[4\varepsilon z-\lambda^{2}\left(1+\theta \right)]}^{2}}>0$, $\frac{\partial w^{nm}}{\partial \theta }=\frac{4z\lambda^{2}\left(D-\varepsilon c\right)}{{[4\varepsilon z-\lambda^{2}\left(1+\theta \right)]}^{2}}>0$,

$$\frac{\partial p^{nm}}{\partial \theta }=\frac{6z\lambda^{2}\left(D-\varepsilon c\right)}{{[4\varepsilon z-\lambda^{2}\left(1+\theta \right)]}^{2}}>0,\frac{\partial q^{nm}}{\partial \theta }=\frac{2\varepsilon z\lambda^{2}\left(D-\varepsilon c\right)}{{[4\varepsilon z-\lambda^{2}\left(1+\theta \right)]}^{2}}>0\text{;}$$

$$\frac{\partial e^{nr}}{\partial \theta }=\frac{\lambda \left(D-\varepsilon c\right)[8\varepsilon z+\lambda^{2}\left(1+\theta \right)]}{\left(1+\theta \right){[8\varepsilon z-\lambda^{2}\left(1+\theta \right)]}^{2}}>0,\frac{\partial w^{nr}}{\partial \theta }=\frac{4z\lambda^{2}\left(D-\varepsilon c\right)}{{[8\varepsilon z-\lambda^{2}\left(1+\theta \right)]}^{2}}>0$$

$$\frac{\partial p^{nr}}{\partial \theta }=\frac{12z\lambda^{2}\left(D-\varepsilon c\right)}{{[8\varepsilon z-\lambda^{2}\left(1+\theta \right)]}^{2}}>0,\frac{\partial q^{nr}}{\partial \theta }=\frac{4\varepsilon z\lambda^{2}\left(D-\varepsilon c\right)}{{[8\varepsilon z-\lambda^{2}\left(1+\theta \right)]}^{2}}>0$$

(2) $\frac{\partial e^{sm}}{\partial \theta }=\frac{\lambda \left(D-\varepsilon c\right)[2\varepsilon z\left(2-\varphi_{m}\right)+\lambda^{2}\left(1+\theta \right)]}{{\left(1+\theta \right)[2\varepsilon z\left(2-\varphi_{m}\right)-\lambda^{2}\left(1+\theta \right)]}^{2}}>0$, $\frac{\partial w^{sm}}{\partial \theta }=\frac{4z\lambda^{2}\left(1-\varphi_{m}\right)\left(D-\varepsilon c\right)}{{[2\varepsilon z\left(2-\varphi_{m}\right)-\lambda^{2}\left(1+\theta \right)]}^{2}}>0$,

$$\frac{\partial p^{sm}}{\partial \theta }=\frac{2z\lambda^{2}\left(3-{2\varphi }_{m}\right)\left(D-\varepsilon c\right)}{{[2\varepsilon z\left(2-\varphi_{m}\right)-\lambda^{2}\left(1+\theta \right)]}^{2}}>0,\frac{\partial q^{sm}}{\partial \theta }=\frac{2\varepsilon z\lambda^{2}\left(D-\varepsilon c\right)}{{[2\varepsilon z\left(2-\varphi_{m}\right)-\lambda^{2}\left(1+\theta \right)]}^{2}}>0\text{;}$$

$$\frac{\partial e^{sr}}{\partial \theta }=\frac{\lambda \left(D-\varepsilon c\right)[2\varepsilon z{\left(2-\varphi_{r}\right)}^{2}+\lambda^{2}\left(1+\theta \right)]}{{[2\varepsilon z{\left(2-\varphi_{r}\right)}^{2}-\lambda^{2}\left(1+\theta \right)]}^{2}}>0,\frac{\partial w^{sr}}{\partial \theta }=\frac{2z\lambda^{2}\left(2-\varphi_{r}\right)\left(D-\varepsilon c\right)}{{[2\varepsilon z{\left(2-\varphi_{r}\right)}^{2}-\lambda^{2}\left(1+\theta \right)]}^{2}}>0\text{,}$$

$$\frac{\partial p^{sr}}{\partial \theta }=\frac{2z\lambda^{2}\left(3-2\varphi_{r}\right)\left(2-\varphi_{r}\right)\left(D-\varepsilon c\right)}{{[2\varepsilon z{\left(2-\varphi_{r}\right)}^{2}-\lambda^{2}\left(1+\theta \right)]}^{2}}>0,\frac{\partial q^{sr}}{\partial \theta }=\frac{2\varepsilon z\lambda^{2}\left(2-\varphi_{r}\right)\left(D-\varepsilon c\right)}{{[2\varepsilon z{\left(2-\varphi_{r}\right)}^{2}-\lambda^{2}\left(1+\theta \right)]}^{2}}>0\text{.}$$

Proof of Proposition 7.

(1) $\frac{\partial \Pi_{m}^{nm}\left(w,e\right)}{\partial \theta }=\frac{z{\left(D-\varepsilon c\right)}^{2}\lambda^{2}}{{[4\varepsilon z-\lambda^{2}\left(1+\theta \right)]}^{2}}>0$, $\frac{\partial \Pi_{r}^{nm}\left(P\right)}{\partial \theta }=\frac{4\varepsilon z^{2}\lambda^{2}{\left(D-\varepsilon c\right)}^{2}}{{[4\varepsilon z-\lambda^{2}\left(1+\theta \right)]}^{3}}>0$,

$$\frac{\partial \Pi_{m}^{nr}\left(w,e\right)}{\partial \theta }=\frac{z{\left(D-\varepsilon c\right)}^{2}\lambda^{2}}{{[8\varepsilon z-\lambda^{2}\left(1+\theta \right)]}^{2}}>0,\frac{\partial \Pi_{r}^{nr}\left(P\right)}{\partial \theta }=\frac{32\varepsilon z^{2}\lambda^{2}{\left(D-\varepsilon c\right)}^{2}}{{[8\varepsilon z-\lambda^{2}\left(1+\theta \right)]}^{3}}>0\text{.}$$

(2) $\frac{\partial \Pi_{m}^{sm}\left(w,e\right)}{\partial \theta }=\frac{z{\left(D-\varepsilon c\right)}^{2}\lambda^{2}}{{[2\varepsilon z\left(2-\varphi_{m}\right)-\lambda^{2}\left(1+\theta \right)]}^{2}}>0,\frac{\partial \Pi_{r}^{sm}\left(P\right)}{\partial \theta }=\frac{2\varepsilon z^{2}\lambda^{2}{\left(1-\varphi_{m}\right)\left(D-\varepsilon c\right)}^{2}}{\left(1+\theta \right){[2\varepsilon z\left(2-\varphi_{m}\right)-\lambda^{2}\left(1+\theta \right)]}^{4}}>0$,

$$\frac{\partial \Pi_{m}^{sr}\left(w,e\right)}{\partial \theta }=\frac{z{\left(1-\varphi_{r}\right)\left(D-\varepsilon c\right)}^{2}\lambda^{2}}{{[2\varepsilon z{\left(2-\varphi_{r}\right)}^{2}-\lambda^{2}\left(1+\theta \right)]}^{2}}>0\text{,}$$

$$\frac{\partial \Pi_{r}^{sr}\left(P\right)}{\partial \theta }=\frac{z{\left(D-\varepsilon c\right)}^{2}\lambda^{2}[2\varepsilon z{\left(2-\varphi_{r}\right)}^{2}\left(4-3\varphi_{r}\right)-\varphi_{r}\lambda^{2}\left(1+\theta \right)]}{{[2\varepsilon z{\left(2-\varphi_{r}\right)}^{2}-\lambda^{2}\left(1+\theta \right)]}^{3}}>0\text{,}$$

Given $2\varepsilon z{\left(2-\varphi_{r}\right)}^{2}-\lambda^{2}\left(1+\theta \right)>0$,from $2\varepsilon z{\left(2-\varphi_{r}\right)}^{2}\left(4-3\varphi_{r}\right)-\varphi_{r}\lambda^{2}\left(1+\theta \right)-2\varepsilon z{\left(2-\varphi_{r}\right)}^{2}+\lambda^{2}\left(1+\theta \right)=\left(1-\varphi_{r}\right)\left[6\varepsilon z{\left(2-\varphi_{r}\right)}^{2}+\lambda^{2}\left(1+\theta \right)\right]>0$, we obtain $2\varepsilon z{\left(2-\varphi_{r}\right)}^{2}\left(4-3\varphi_{r}\right)-\varphi_{r}\lambda^{2}\left(1+\theta \right)>0$, so $\frac{\partial \Pi_{r}^{sr}\left(P\right)}{\partial \theta }>0$.

Proof of Proposition 8.

(1) Given $0<\varphi_{m}<50\%$, then $\frac{\partial \theta }{\partial \varphi_{m}}=\frac{\varepsilon z}{\lambda^{2}}>0$.
(2) Given ${0<\varphi }_{r}<50\%$, then $\frac{\partial \theta }{\partial \varphi_{r}}=\frac{\lambda^{2}\left(1+\theta \right)-2\varepsilon z\left(2-\varphi_{r}\right)\varphi_{r}}{2\left(1-\varphi_{r}\right)\lambda^{2}}$. We get (i) when $0<u_{e}<\frac{3}{4}$, if ${0<\varphi }_{r}<\varphi_{r3}$, then $\frac{\partial \theta }{\partial \varphi_{r}}>0$; if ${\varphi_{r3}\leq \varphi }_{r}<50\%$, then $\frac{\partial \theta }{\partial \varphi_{r}}\leq 0$. (ii) when $\frac{3}{4}\leq u_{e}<\frac{9}{4}$, the manufacturer technology spillover rate θ will increase, i.e. $\frac{\partial \theta }{\partial \varphi_{r}}>0$, where $\varphi_{r3}=1-\sqrt{1-u_{e}}$.

## Appendix D

### Proof of Lemma 1

From $\Pi_{t}^{I}\left(e,p\right)$, we obtain $\frac{\partial^{2}\Pi_{t}^{I}\left(e,p\right)}{\partial p^{2}}=-2\varepsilon <0$, so $\Pi_{t}^{I}\left(e,p\right)$ is a concave function of p. We let $\frac{\partial \Pi_{t}^{I}\left(e,p\right)}{\partial p}=0$ and get $p^{I}\left(e\right)=\frac{D+\varepsilon c+\lambda \left(1+\theta \right)e}{2\varepsilon }$. Replacing $p^{I}\left(e\right)$ in $\Pi_{t}^{I}\left(e,p\right)$, we obtain $\frac{\partial^{2}\Pi_{t}^{I}\left(e,p\right)}{\partial e^{2}}=\frac{\lambda^{2}{\left(1+\theta \right)}^{2}}{2\varepsilon }-z\left(1+\theta \right)$. To ensure that the model has a solution, we assume $\frac{\lambda^{2}{\left(1+\theta \right)}^{2}}{2\varepsilon }-z\left(1+\theta \right)<0$, that is $\frac{\lambda^{2}\left(1+\theta \right)}{2\varepsilon z}<1$, then we have $0<u_{e}<1$. We let $\frac{\partial \Pi_{t}^{I}\left(e,p\right)}{\partial e}=0$, and we obtain $e^{I}=\frac{\left(D-\varepsilon c\right)\lambda }{2\varepsilon z-\lambda^{2}\left(1+\theta \right)}$. Substitute $e^{I}$ into $p^{I}\left(e\right)$ to get $p^{I}=c+\frac{z\left(D-\varepsilon c\right)}{2\varepsilon z-\lambda^{2}\left(1+\theta \right)}$. In addition, we can get the following results: $q^{I}=\frac{\varepsilon z\left(D-\varepsilon c\right)}{2\varepsilon z-\lambda^{2}\left(1+\theta \right)}$, $\Pi_{t}^{I}\left(e,p\right)=\frac{z{\left(D-\varepsilon c\right)}^{2}}{2\left[2\varepsilon z-\lambda^{2}\left(1+\theta \right)\right]}$.

### Proof of Corollary 1

(1) In the MD model, $\Pi_{t}^{I}\left(e,p\right)-\Pi_{t}^{sm}\left(w,e,p\right)=\frac{4\varepsilon^{2}z^{3}{\left(1-\varphi_{m}\right)}^{2}{\left(D-\varepsilon c\right)}^{2}}{4\left[2\varepsilon z-\lambda^{2}\left(1+\theta \right)\right]{[2\varepsilon z\left(2-\varphi_{m}\right)-\lambda^{2}\left(1+\theta \right)]}^{2}}>0$.
(2) In the RD model,

$$\Pi_{t}^{I}\left(e,p\right)-\Pi_{t}^{sr}\left(w,e,p\right)=\frac{z{\left(D-\varepsilon c\right)}^{2}\{4\varepsilon^{2}z^{2}{\left(2-\varphi_{r}\right)}^{2}\left(1+\varphi_{r}^{2}\right)+2\varepsilon z\lambda^{2}\left(1+\theta \right)[1+{\left(2-\varphi_{r}\right)}^{2}\left(1-2\varphi_{r}\right)]\}}{4\left[2\varepsilon z-\lambda^{2}\left(1+\theta \right)\right]{[2\varepsilon z{\left(2-\varphi_{r}\right)}^{2}-\lambda^{2}\left(1+\theta \right)]}^{2}}>0\text{.}$$

Proof of Proposition 9.

(1) From $\Pi_{r}^{MT}\left(p\right)$, we obtain $\frac{\partial \Pi_{r}^{MT}\left(p\right)}{\partial p}=\left(1-\varphi_{m}\right)\left[D-\varepsilon p+\lambda \left(1+\theta \right)e\right]-\varepsilon \left(1-\varphi_{m}\right)\left(p-w\right)$ and $\frac{\partial^{2}\Pi_{r}^{MT}\left(p\right)}{\partial p^{2}}=-2\varepsilon \left(1-\varphi_{m}\right)<0$, so $\Pi_{r}^{MT}\left(p\right)$ is a concave function of p. We let $\frac{\partial \Pi_{r}^{MT}\left(p\right)}{\partial p}=0$, and we obtain D+λ(1+θ)e−2εp+εw=0. To coordinate the supply chain, we substitute $e^{sm}=e^{I}$, $p^{sm}=p^{I}$ into the above formula. We make $\varepsilon \left(w-c\right)\left[2\varepsilon z-\lambda^{2}\left(1+\theta \right)\right]=0$, hence $w^{sm}=c$.

In the MD model, the manufacturer gains additional profit from supply chain coordination as the supply chain leader. Regarding the retailer, $\Pi_{r}^{MT}\left(p^{I}\right)-\Pi_{r}^{sm}\left(p^{sm}\right)=\frac{4\varepsilon^{2}z^{3}{\left(1-\varphi_{m}\right)}^{2}{\left(D-\varepsilon c\right)}^{2}\left[\left(3-\varphi_{m}\right)\varepsilon z-\lambda^{2}\left(1+\theta \right)\right]}{{\left[2\varepsilon z-\lambda^{2}\left(1+\theta \right)\right]}^{2}{\left[2\varepsilon z\left(2-\varphi_{m}\right)-\lambda^{2}\left(1+\theta \right)\right]}^{2}}-M^{sm}=0$. Then we obtain $M^{sm}=\frac{4\varepsilon^{2}z^{3}{\left(1-\varphi_{m}\right)}^{2}{\left(D-\varepsilon c\right)}^{2}\left[\left(3-\varphi_{m}\right)\varepsilon z-\lambda^{2}\left(1+\theta \right)\right]}{{\left[2\varepsilon z-\lambda^{2}\left(1+\theta \right)\right]}^{2}{\left[2\varepsilon z\left(2-\varphi_{m}\right)-\lambda^{2}\left(1+\theta \right)\right]}^{2}}$. Regarding the manufacturer, $\Pi_{m}^{MT}\left(w^{I},e^{I}\right)-\Pi_{m}^{sm}\left(w^{sm},e^{sm}\right)=\frac{z{\left(D-\varepsilon c\right)}^{2}\{4\varepsilon^{2}z^{2}\left[2\varepsilon z-\lambda^{2}\left(1+\theta \right)\right]+{\left[2\varepsilon z\left(2-\varphi_{m}\right)-\lambda^{2}\left(1+\theta \right)\right]}^{2}\lambda^{2}\left(1+\theta \right)\}}{2{\left[2\varepsilon z-\lambda^{2}\left(1+\theta \right)\right]}^{2}{\left[2\varepsilon z\left(2-\varphi_{m}\right)-\lambda^{2}\left(1+\theta \right)\right]}^{2}}>0$.

So in the MD model, the supply chain coordination condition is $w^{sm}=c$, the Pareto improvement area is $M^{sm}=\frac{4\varepsilon^{2}z^{3}{\left(1-\varphi_{m}\right)}^{2}{\left(D-\varepsilon c\right)}^{2}\left[\left(3-\varphi_{m}\right)\varepsilon z-\lambda^{2}\left(1+\theta \right)\right]}{{\left[2\varepsilon z-\lambda^{2}\left(1+\theta \right)\right]}^{2}{\left[2\varepsilon z\left(2-\varphi_{m}\right)-\lambda^{2}\left(1+\theta \right)\right]}^{2}}$.

(2) From $\Pi_{r}^{RT}\left(p\right)$, we obtain $\frac{\partial \Pi_{r}^{RT}\left(p\right)}{\partial p}=D-\varepsilon p+\lambda \left(1+\theta \right)e-\varepsilon \left(p-w\right)-\varepsilon \varphi_{r}\left(w-c\right)$ and $\frac{\partial^{2}\Pi_{r}^{RT}\left(p\right)}{\partial p^{2}}=-2\varepsilon <0$, so $\Pi_{r}^{RT}\left(p\right)$ is a concave function of p. Let $\frac{\partial \Pi_{r}^{RT}\left(p\right)}{\partial p}=0$, we get $D-2\varepsilon p+\lambda \left(1+\theta \right)e+\left(1-\varphi_{r}\right)\varepsilon w+\varphi_{r}\varepsilon c=0$. To coordinate the supply chain, we substitute $e^{sr}=e^{I}$, $p^{sr}=p^{I}$ into the above formula and we obtain $\left(1-\varphi_{r}\right)\varepsilon \left(w-c\right)=0$, hence $w^{sr}=c$.

In the RD model, the retailer gains additional profit from supply chain coordination as the supply chain leader. Regarding the manufacturer, $\Pi_{m}^{MT}\left(w^{I},e^{I}\right)-\Pi_{m}^{sr}\left(w^{I},e^{I}\right)=M^{sr}-\frac{\left(1-\varphi_{r}\right)z{\left(D-\varepsilon c\right)}^{2}\lambda^{2}\left(1+\theta \right)}{2{\left[2\varepsilon z-\lambda^{2}\left(1+\theta \right)\right]}^{2}}-\frac{\left(1-\varphi_{r}\right)z{\left(D-\varepsilon c\right)}^{2}}{2\left[2\varepsilon z{\left(2-\varphi_{r}\right)}^{2}-\lambda^{2}\left(1+\theta \right)\right]}=0$, then we obtain $M^{sr}=\frac{\left(1-\varphi_{r}\right)z{\left(D-\varepsilon c\right)}^{2}\lambda^{2}\left(1+\theta \right)}{2{\left[2\varepsilon z-\lambda^{2}\left(1+\theta \right)\right]}^{2}}+\frac{\left(1-\varphi_{r}\right)z{\left(D-\varepsilon c\right)}^{2}}{2\left[2\varepsilon z{\left(2-\varphi_{r}\right)}^{2}-\lambda^{2}\left(1+\theta \right)\right]}$. Regarding the retailer, $\Pi_{r}^{MT}\left(p^{I}\right)-\Pi_{r}^{sm}\left(p^{sm}\right)=\frac{z{\left(D-\varepsilon c\right)}^{2}\{4\varepsilon^{2}z^{2}{\left(2-\varphi_{r}\right)}^{2}{\left(1-\varphi_{r}\right)}^{2}+2\varepsilon z{\left(2-\varphi_{r}\right)}^{2}\left(1-2\varphi_{r}\right)\lambda^{2}\left(1+\theta \right)+2\varepsilon z\lambda^{2}\left(1+\theta \right)\}}{2\left(1+\theta \right){\left[2\varepsilon z-\lambda^{2}\left(1+\theta \right)\right]}^{2}{\left[2\varepsilon z{\left(2-\varphi_{r}\right)}^{2}-\lambda^{2}\left(1+\theta \right)\right]}^{2}}>0$.

### Proof of Lemma 2

(1) In the MD model,

$$\frac{\partial M^{sm}}{\partial \varphi_{m}}=\frac{-\varepsilon z^{2}{\left(D-\varepsilon c\right)}^{2}\left\{{\left[2\varepsilon z\left(2-\varphi_{m}\right)-\lambda^{2}\left(1+\theta \right)\right]}^{3}-{\left[2\varepsilon z-\lambda^{2}\left(1+\theta \right)\right]}^{2}\left[2\varepsilon z\varphi_{m}-\lambda^{2}\left(1+\theta \right)\right]\right\}}{{\left[2\varepsilon z-\lambda^{2}\left(1+\theta \right)\right]}^{2}{\left[2\varepsilon z\left(2-\varphi_{m}\right)-\lambda^{2}\left(1+\theta \right)\right]}^{3}}\text{.}$$

From ${\left[2\varepsilon z\left(2-\varphi_{m}\right)-\lambda^{2}\left(1+\theta \right)\right]}^{2}>{\left[2\varepsilon z-\lambda^{2}\left(1+\theta \right)\right]}^{2}$ and $\left[2\varepsilon z\left(2-\varphi_{m}\right)-\lambda^{2}\left(1+\theta \right)\right]>\left[2\varepsilon z\varphi_{m}-\lambda^{2}\left(1+\theta \right)\right]$, we obtain $\frac{\partial M^{sm}}{\partial \varphi_{m}}<0$.

(2) In the RD model,

$$\frac{\partial M^{sr}}{\partial \varphi_{r}}=\frac{-z{\left(D-\varepsilon c\right)}^{2}\left\{{\left[2\varepsilon z{\left(2-\varphi_{r}\right)}^{2}-\lambda^{2}\left(1+\theta \right)\right]}^{2}\lambda^{2}\left(1+\theta \right)+{\left[2\varepsilon z-\lambda^{2}\left(1+\theta \right)\right]}^{2}\left[2\varepsilon z\left(2-\varphi_{r}\right)\varphi_{r}-\lambda^{2}\left(1+\theta \right)\right]\right\}}{{2\left[2\varepsilon z-\lambda^{2}\left(1+\theta \right)\right]}^{2}{\left[2\varepsilon z{\left(2-\varphi_{r}\right)}^{2}-\lambda^{2}\left(1+\theta \right)\right]}^{2}}\text{.}$$

Category discussion.

(a) when $2\varepsilon z\left(2-\varphi_{r}\right)\varphi_{r}-\lambda^{2}\left(1+\theta \right)\geq 0$, then $\frac{\partial M^{sr}}{\partial \varphi_{r}}<0$;
(b) when $2\varepsilon z\left(2-\varphi_{r}\right)\varphi_{r}-\lambda^{2}\left(1+\theta \right)<0$,

$$\frac{\partial M^{sr}}{\partial \varphi_{r}}=\frac{-z{\left(D-\varepsilon c\right)}^{2}\{{\left[2\varepsilon z{\left(2-\varphi_{r}\right)}^{2}-\lambda^{2}\left(1+\theta \right)\right]}^{2}\lambda^{2}\left(1+\theta \right)}{-{\left[2\varepsilon z-\lambda^{2}\left(1+\theta \right)\right]}^{2}\left[\lambda^{2}\left(1+\theta \right)-2\varepsilon z\left(2-\varphi_{r}\right)\varphi_{r}\right]\}{2\left[2\varepsilon z-\lambda^{2}\left(1+\theta \right)\right]}^{2}{\left[2\varepsilon z{\left(2-\varphi_{r}\right)}^{2}-\lambda^{2}\left(1+\theta \right)\right]}^{2}}$$

From ${\left[2\varepsilon z{\left(2-\varphi_{r}\right)}^{2}-\lambda^{2}\left(1+\theta \right)\right]}^{2}>{\left[2\varepsilon z-\lambda^{2}\left(1+\theta \right)\right]}^{2}$ and $\lambda^{2}\left(1+\theta \right)>\lambda^{2}\left(1+\theta \right)-2\varepsilon z\left(2-\varphi_{r}\right)\varphi_{r}$, we obtain ${\left[2\varepsilon z{\left(2-\varphi_{r}\right)}^{2}-\lambda^{2}\left(1+\theta \right)\right]}^{2}\lambda^{2}\left(1+\theta \right)-{\left[2\varepsilon z-\lambda^{2}\left(1+\theta \right)\right]}^{2}\left[\lambda^{2}\left(1+\theta \right)-2\varepsilon z\left(2-\varphi_{r}\right)\varphi_{r}\right]>0$, then $\frac{\partial M^{sr}}{\partial \varphi_{r}}<0$.

In summary, we obtain $\frac{\partial M^{sr}}{\partial \varphi_{r}}<0$.

## Footnotes

1 http://newsroom.accenture.com/article_display.cfm?article_id=4801.

2 http://www.carbontrust.com/news/2011/07/consumer-demand-for-lower-carbon-lifestyles-is-putting-pressure-on-business.

3 https://www.sohu.com/a/329594561_161795.

4 https://www.haier.com/about_haier/xinwen/20220902_201249.shtml.

5 https://www.txp-life.com/.

6 Annual report of the company.

## References

[1] Hong Z., Guo X. (2019). Green product supply contracts considering environmental responsibilities. Omega.

[2] Kefalew T., Tilinti B., Betemariyam M. (2021). The potential of biogas technology in fuelwood saving and carbon emission reduction in Central Rift Valley, Ethiopia. Heliyon.

[3] Peng W., Xin B., Xie L. (2023). Optimal strategies for production plan and carbon emission reduction in a hydrogen supply chain under cap-and-trade policy. Renew. Energy.

[4] Wang C., Wang L., Wang W., Xiong Y., Du C. (2023). Does carbon emission trading policy promote the corporate technological innovation? Empirical evidence from China's high-carbon industries. J. Clean. Prod..

[5] Yang P., Peng S., Benani N., Dong L., Li X., Liu R., Mao G. (2022). An integrated evaluation on China's provincial carbon peak and carbon neutrality. J. Clean. Prod..

[6] Dong C., Liu Q., Shen B. (2019). To be or not to be green? Strategic investment for green product development in a supply chain. Transport. Res. Part E.

[7] Xu J., Chen Y., Bai Q. (2016). A two-echelon sustainable supply chain coordination under cap-and-trade regulation. J. Clean. Prod..

[8] Roels G., Tang C.S. (2017). Win-win capacity allocation contracts in coproduction and codistribution alliances. Manag. Sci..

[9] Cabral L., Pacheco-de-Almeida G. (2019). Alliance formation and firm value. Manag. Sci..

[10] Lechler S., Canzaniello A., Hartmann E. (2019). Assessment sharing intra-industry strategic alliances: Eﬀects on sustainable supplier management within multi-tier supply chains. Int. J. Prod. Econ..

[11] Kale P., Singh H. (2009). Managing strategic alliances: what do we know now, and where do wo go from here?. Acad. Manag. Perspect..

[12] Niu W., Shen H. (2022). Investment in process innovation in supply chains with knowledge spillovers under innovation uncertainty. Eur. J. Oper. Res..

[13] Dyer J.H., Nobeoka K. (2000). Creating and managing a high-performance knowledge-sharing network: the Toyota case. Strat. Manag. J..

[14] Xu L., Luo Y., Pu X. (2023). Information acquisition from data-driven analytics: a perspective of blockchain service in a duopoly market. Comput. Ind. Eng..

[15] Houssam N., Ibrahiem D.M., Sucharita S., El-Aasar K.M., Esily R.R., Sethi N. (2023). Assessing the role of green economy on sustainable development in developing countries. Heliyon.

[16] Shahzad F., Du J., Khan I., Shahbaz M., Murad M., Khan M.A.S. (2020). Untangling the influence of organizational compatibility on green supply chain management efforts to boost organizational performance through information technology capabilities. J. Clean. Prod..

[17] Jin M., Zhang X., Xiong Y., Zhou Y. (2021). Implications of green optimism upon sustainable supply chain management. Eur. J. Oper. Res..

[18] Wang Y., Yu Z., Jin M., Mao J. (2021). Decisions and coordination of retailer-led low-carbon supply chain under altruistic preference. Eur. J. Oper. Res..

[19] Daryanto Y., Wee H.M., Astanti R.D. (2019). Three-echelon supply model considering carbon emission and item deterioration. Transport. Res. Part E.

[20] Luo R., Zhou L., Song Y., Fan T. (2022). Evaluating the impact of carbon tax policy on manufacturing and remanufacturing decisions in a closed-loop supply chain. Int. J. Prod. Econ..

[21] Abbas A., Luo X., Shahzad F., Wattoo M.U. (2023). Optimizing organizational performance in manufacturing: the role of IT capability, green supply chain integration, and green innovation. J. Clean. Prod..

[22] Shahzad F., Du J., Khan I., Wang J. (2022). Decoupling institutional pressure on green supply chain management efforts to boost organizational performance: Moderating impact of big data analytics capabilities. Front. Environ. Sci..

[23] Zhang X., Xiu G., Shahzad F., Duan Y. (2021). Optimal financing strategy in a capital-constrained supply chain with retailer green marketing efforts. Sustainability.

[24] Li X., Du J., Li W., Shahzad F. (2023). Green ambitions: a comprehensive model for enhanced traceability in agricultural product supply chain to ensure quality and safety. J. Clean. Prod..

[25] Zhu C., Du J., Shahzad F., Wattoo M.U. (2022). Environment sustainability is a corporate social responsibility: Measuring the Nexus between sustainable supply chain management, big data analytical capabilities, and organizational performance. Sustainability.

[26] Xu L., Shi J., Chen J., Li D.K., Mclaughlin H.L. (2023). Agency encroachment and information sharing: cooperation and competition in freight forwarding market. Marit. Pol. Manag..

[27] Klassen R.D., Vachon S. (2003). Collaboration and evaluation in the supply chain: the impact on plant-level environmental investment. Prod. Oper. Manag..

[28] Jira C., Toffel M.W. (2013). Engaging supply chains in climate change. Manuf. Serv. Oper. Manag..

[29] Dai R., Zhang J., Tang W. (2017). Cartelization or cost-sharing? Comparison of cooperation models in a green supply chain. J. Clean. Prod..

[30] Hafezalkotob A. (2017). Competition, cooperation, and coopetition of green supply chains under regulations on energy saving levels. Transport. Res. Part E.

[31] Kuiti M.R., Ghosh D., Basu P., Bisi A. (2022). Do cap-and-trade policies drive environmental and social goals in supply chains: strategic decisions, collaboration, and contract choices. Int. J. Prod. Econ..

[32] Lyu B., Yi R., Fan G., Zhang Y. (2023). Stakeholder network for developing open innovation practice of China's manufacturing enterprises. Heliyon.

[33] Flath D. (1989). Vertical integration by means of shareholding interlocks. Int. J. Ind. Organ..

[34] Greenlee P., Raskovich A. (2006). Partial vertical ownership. Eur. Econ. Rev..

[35] Qi A., Ahn H.-S., Sinha A. (2016). Investing in a shared supplier in a competitive market: Stochastic capacity case. Prod. Oper. Manag..

[36] Hunold M., Stahl K. (2016). Passive vertical integration and strategic delegation. Rand J. Econ..

[37] Gaigné C., Latouche K., Turolla S. (2018). Vertical ownership and export performance: firm-level evidence from the food industry. Am. J. Agric. Econ..

[38] Fu H., Ma Y., Cai X. (2018). Downstream firm's investment with equity holding in decentralized assembly systems. Omega.

[39] Zhang X., Xiu G., Shahzad F., Duan C. (2021). The impact of equity financing on the performance of capital-constrained supply chain under consumers' low-carbon preference. Int. J. Environ. Res. Publ. Health.

[40] Chen J., Hu Q., Song J.-S. (2017). Effect of partial cross ownership on supply chain performance. Eur. J. Oper. Res..

[41] Ren D., Guo R., Lan Y., Shang C. (2021). Shareholding strategies for selling green products on online platforms in a two-echelon supply chain. Transport. Res. Part E.

[42] Fu H., Ma Y. (2019). Optimization and coordination of decentralized supply chains with vertical cross-shareholding. Comput. Ind. Eng..

[43] Xia Q., Zhi B., Wang X. (2021). The role of cross-shareholding in the green supply chain: green contribution, power structure and coordination. Int. J. Prod. Econ..

[44] Wang Y., Xiao Y., Yang N. (2014). Improving reliability of a shared supplier with competition and spillovers. Eur. J. Oper. Res..

[45] Wang C.C., Wu A. (2016). Geographical FDI knowledge spillover and innovation of indigenous firms in China. Int. Bus. Rev..

[46] Ge Z., Hu Q., Xia Y. (2014). Firms' R&D cooperation behavior in a supply chain. Prod. Oper. Manag..

[47] Byun S.K., Oh J.M., Xia H. (2021). Incremental vs. breakthrough innovation: the role of technology spillovers. Manag. Sci..

[48] Chen X., Wang X., Zhou M. (2019). Firms' green R&D cooperation behaviour in a supply chain: technological spillover, power and coordination. Int. J. Prod. Econ..

[49] Niu W., Shen H. (2022). Investment in process innovation in supply chains with knowledge spillovers under innovation uncertainty. Eur. J. Oper. Res..

[50] Yao D.Q., Liu J.J. (2005). Competitive pricing of mixed retail and e-tail distribution channels. Omega.

[51] Agi M.A.N., Yan X. (2020). Greening products in a supply chain under market segmentation and different channel power structures. Int. J. Prod. Econ..

[52] Pan K., Lai K.K., Leung S.C.H., Di X. (2010). Revenue-sharing versus wholesale price mechanisms under different channel power structures. Eur. J. Oper. Res..

[53] Meng X., Yao Z., Nie J., Zhao Y., Li Z. (2018). Low-carbon product selection with carbon tax and competition: effects of the power structure. Int. J. Prod. Econ..

[54] Hu Y., Qu S., Li G., Sethi S.P. (2021). Power structure and channel integration strategy for online retailers. Eur. J. Oper. Res..

[55] Li M.M., Mizuno S.j. (2022). Dynamic pricing and inventory management of a dual-channel supply chain under different power structures. Eur. J. Oper. Res..

[56] Veldman J., Gaalman G.J.C. (2015). Competitive investments in cost reducing process improvement: the role of managerial incentives and spillover learning. Int. J. Prod. Econ..

[57] Yue L., Miao J., Ahmad F., Draz M.U., Guan H., Chandio A.A., Abid N. (2022). Investigating the role of international industrial transfer and technology spillovers on industrial land production efficiency: fresh evidence based on Directional Distance Functions for Chinese provinces. J. Clean. Prod..

[58] Chen X., Wang X., Chan H.K. (2017). Manufacturer and retailer coordination for environmental and economic competitiveness: a power perspective. Transport. Res. Part E.

[59] Tiep Le T., Vinh Vo X., Venkatesh V.G. (2022). Role of green innovation and supply chain management in driving sustainable corporate performance. J. Clean. Prod..

[60] He X., Khouja M. (2011). Pareto analysis of supply chain contracts under satisficing objectives. Eur. J. Oper. Res..

